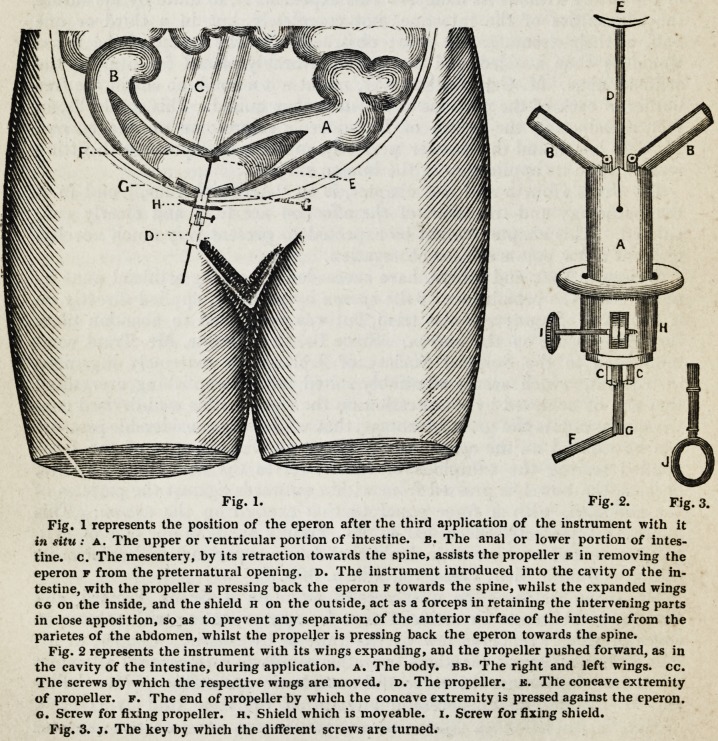# An Experimental and Critical Inquiry into the Nature and Treatment of Wounds of the Intestines; Illustrated by Engravings

**Published:** 1847-01

**Authors:** 


### BRITISH AND FOREIGN

### MEDICAL REVIEW,

FOR JANUARY, 1847.

### PART FIRST.

### Analytical and Critical Reviews.

### Art. I.

1. *An Experimental and Critical Inquiry into the Nature and Treatment of Wounds of the Intestines; illustrated by Engravings.* By Samuel D. Gross, M.D., Professor of Surgery in the Louisville Medical Institute, Surgeon to the Louisville Marine Hospital, &c.—*Louisville*, 1843. 8vo, pp. 219.
2. *Recherches sur l'Emploi d'un Nouveau Procédé de Suture contre les Divisions de l'Intestin et sur la possibilité de l'Adossement de cet Organe avec lui-même dans certaines Blessures.* Par J. A. Gely (de Nantes), M.D. Mémoire addressé à l'Académie Royale de Médecine.

*Researches on the Application of a New Method of Suture in Wounds of the Intestines, and on the possibility of inflecting the Intestine on itself in certain Wounds of that Organ.* By J. A. Gely, M.D., of Nantes. Memoir presented to the Royal Academy of Medicine of Paris.—8vo, pp. 84.

3. *Die Operative Chirurgie.* Von Johann F. Dieffenbach. Erster Band. Capitel LXII. *Operation des widernaturlichen Afters.—Leipzig*, 1845.

Dr. Gross thinks it necessary to make an apology for the publication of a work on Wounds of the Intestines, and pleads "that our systematic treatises on surgery unfortunately contain little, if anything, that is worthy of reliance; they enter into no details, and some of them do not even allude to the subject." This is unquestionably to a great extent true; and it might have been added that there are few subjects in surgery respecting which both systematic and other writers differ more widely. Dr. Gross needs to make no apology for the present publication; his attention has been directed towards a most proper and legitimate field of research, and his work, therefore, claims an examination which we regret circumstances have prevented us sooner giving to it.

Dr. Gross, with reason, dates the commencement of scientific knowledge respecting wounds of the intestines from the publication of Mr. Travers's excellent work in 1812, stating that he "did not, like his predecessors, limit his inquiries to the human subject, but extended them to the inferior animals;" at the same time Dr. Gross claims priority in this respect to a certain extent for Dr. Thomas Smith. We may just observe that Mr. Travers refers to Dr. Smith's experiments, and that it is not quite accurate to imply that either Mr. Travers or Dr. Smith had no predecessors in this branch of experimental inquiry. Mr. Travers very fully notices the antecedent experiments of Wm. Cowper, Mr. Skipton, Dr. Thomson, and Sir A. Cooper, and alludes to some by Mœbius and Louis. De Blegny, also, in 1682, Dr. Wallis in 1695, Vogel in 1704, Brunner in 1722, Schlichting in 1742, Mr. Watson in 1790, and doubtless others of whom we are ignorant, published experiments and facts respecting wounds of the intestines of animals. The value of those experiments, however, taken even in the aggregate, was but trifling. De Blegny, Brunner, Cowper, and Schlichting did not even dissect the animals they experimented on; and to Mr. Travers unquestionably belongs the merit of having first systematically investigated the subject of wounds of the intestines, and of having put us in possession of any precise and definite knowledge respecting the process whereby such wounds are repaired, whether spontaneously, or with the intervention of art.

Dr. Gross's first chapter, on the "Nature of wounds of the intestines," is divided into four sections, in which he considers the "structure of the alimentary canal," the "nature and extent of the peritoneal cavity," the "symptoms, diagnosis, and prognosis" of wounds of the intestines, and, finally, "their mode of reparation." As regards the three former sections, a few points only require to be adverted to; but the last section demands a more careful examination, which, however, we can most conveniently give to it when considering the chapter on treatment.

Wiseman, La Motte, Garengeot, and, we believe, the older surgeons generally, held that an instrument might traverse the abdomen without injuring its contents, and many modern surgeons are of the same opinion. Dupuytren, for example, states that such an event occurs "very frequently." (Traité des Bless. par Armes de Guerre, t. ii, p. 428.) With this opinion Dr. Gross agrees, while Mr. Travers, as our readers doubtless are aware, strongly maintains the opposite doctrine. Dr. Gross supports his opinion by appealing to the well-known cases recorded by Wiseman, La Motte, Garengeot, Hennen, and others, in which patients recovered rapidly, and without a bad symptom, after the abdomen had been transfixed, and also by adducing, "as strikingly illustrative of the manner in which the intestines glide away from the edge of the knife," certain cases of very extensive wounds of the abdominal parietes, in which the intestines protruded without being injured. As to this latter class of cases, they are quite beside the question, and prove nothing in favour of Dr. Gross's position, or against that of Mr. Travers. The former class of cases, however, are more to the purpose, but they are not conclusive. With respect to them, Dr. Gross, in common with Wiseman, &c., infers the absence of wound of the bowel from the absence of symptoms indicating its occurrence; but that such an inference is unwarranted sufficiently appears from experiments and cases in the human subject, so well known that we shall not occupy space by referring to them. There are, however, some cases on record, which prove that an instrument can pass among the mass of hollow viscera without wounding them. Dr. Gross refers to one inconclusive case recorded by La Motte, in which the patient recovered; but he does not refer to two other cases of fatal sword wounds of the abdomen, also recorded by La Motte: in one of which, on dissection, the vena cava, in the other, the vena cava and the aorta, were wounded, but in both the intestines were uninjured. It may be remarked that Mr. Travers refers to those two cases when treating of hemorrhage into the abdomen, and says, "in neither instance had the sword wounded the viscera" (An Inquiry, &c., p. 72); yet he elsewhere denies the fact which he admits in the words just quoted. Those two are, so far as we know, the only conclusive cases in point recorded when Mr. Travers, or even when Dr. Gross, wrote; but some which go to establish the same fact have since been published. Mr. South, in his recent translation of Chelius's Surgery (Part v, p. 458), gives an analogous case, in which an iron spike traversed the abdomen, tore the right common iliac vein and deeply indented the third lumbar vertebra without injuring any of the viscera. In the 'Experience' (No. 373, Aug. 1844) we find the following remarkable case: a woman, five months pregnant, fell from a tree on a wooden stake, which entered the inner and back part of the left thigh, and could be felt through the soft parts at the external border of the left quadratus lumborum muscle passing up beneath the false ribs. As the stake had broken off deep in the wound, and could not, therefore, be withdrawn from below, M. Scaruffi cut down on it in the lumbar region, opened the peritoneum sufficiently to admit the hand, and extracted a piece of wood 8½ inches long; his hand was in contact with the intestines, which certainly do not appear to have been wounded; the woman, we may add, perfectly recovered. The case recorded by Mr. Hennen, in which a ramrod penetrated the abdomen from before backwards, and became firmly impacted in the spine, has been very frequently quoted by writers. We suspect, but cannot be certain, that the following supplies the sequel of that case; but be that as it may, it bears on the point under consideration. Dr. Gilkrest mentioned at the Westminster Medical Society (Lancet, 1832, p. 147) a case, if not the same, precisely similar to that mentioned by Hennen, in which the patient, shortly after his recovery, was drowned, and no injury of any of the viscera could be detected on the most minute examination. If this latter case is excepted to on the grounds that a cicatrix might have been overlooked, which, considering the nature of the projectile (a ramrod), we think quite improbable, the preceding cases sufficiently establish that an instrument can penetrate between the intestinal convolutions without wounding them; and, indeed, we think the fact is proved by one of Mr. Travers's own experiments (Exp. D, p. 54), in which, a month after having pushed a catlin to the shoulder in the abdomen of a dog, he could not detect any sign of the intestine having been wounded; whence, instead of the more obvious inference that the bowel had escaped injury, Mr. Travers concludes that a simple incision in the intestine may unite *per se* and escape detection.

We have already admitted that the mere fact of absence of symptoms after deeply penetrating wounds of the abdomen affords no evidence that the viscera have escaped intact; but as the fact is certainly sometimes so, it seems not out of place to here mention some of the more recently recorded cases of this very remarkable accident. Mr. Ellis (Lancet, 1834-5, vol. ii, p. 756) mentions a case of attempted suicide, in which a sword entered near the navel, and protruded through the integuments very near the spinal column; in a few days the patient recovered, having manifested scarcely a symptom of injury. M. Roy records a case in which an iron spit entered two inches to the right side of the navel, and was so firmly impacted in the bones of the pelvis near the sacro-iliac symphysis, that on endeavouring to extract it while the patient (a boy) lay on his back, he was lifted from the ground; by a little management the instrument was extracted unbroken, and recovery was uninterrupted by a bad symptom. As the first stool, which was passed on the ninth day, was flattened or riband-like, M. Roy thinks this circumstance may probably indicate temporary contraction of the intestine at some point where it had been perforated. (Gaz. Méd. de Paris, 1843, p. 708.) In the same Journal (1845, p. 313) we find the case of a child, aged 14, who fell on an iron rod used by silk-winders, which passed in at the fold of the left buttock, about 2½ fingers' breadth from the anus, and came out a little below, and to the right side of the umbilicus. The boy was walking down stairs when he was met by his mother, who withdrew the instrument; but the circumstances of the wound were fully ascertained by Dr. Bessem, who discharged him from hospital on the twentieth day, without any symptom indicating injury of the intestine having ensued.

Fæcal effusion is, in the opinion of Mr. Travers, and of almost all surgeons since he wrote, an unusual result of penetrating wounds of the abdomen, which can scarcely indeed occur unless "the gut be full and the wound extensive," or unless the escape of fæces is favoured by the extravasation of a considerable quantity of blood, or of air within the abdomen. Dr. Gross regrets that Mr. Travers, "in the experiments which he instituted to illustrate this branch of the subject, as well as in the cases which he has adduced from his own and the practice of others, has not specified the size of the lesion—a matter of such paramount importance that it is only surprising how it could have been overlooked" (p. 11); and he maintains, from the result of eight experiments performed by himself, that extravasation occurs "very frequently, and with great readiness," that it "almost always, if not invariably," follows "wounds of the bowel to the extent of six lines, whether transverse, oblique, or longitudinal," but that it does not in the majority of cases result from wounds not exceeding four lines in length (pp. 9-10). Dr. Gross's experiments on this point are, however, open to the serious, indeed insuperable objection, that the intestine was displaced from the abdomen, and returned after having been wounded; a circumstance which, as Mr. Travers specially observes, is much more favorable to fæcal effusion than when the gut has been wounded in situ. It is indeed impossible, by experiments so conducted, to determine within any tolerable degree of approximation, the extent of wound that is likely to be followed by fæcal effusion, when the bowel has been opened without prolapsing; and so far as our knowledge at present goes, the occurrence of such effusion seems to be governed much more by accidental concomitant circumstances, such as fulness of the gut, &c., mentioned by all writers on the subject, than by the mere extent of the wound. Dr. Gross says that Mr. Travers's object "appears to have been, not so much to deduce from his experiments on this point any practical precept in reference to the management of" wounds of the intestines, "as to show that the apprehension of intestinal effusion in penetrating wounds of the abdomen is, in the majority of cases, without" foundation (pp. 11-12); but when Mr. Travers wrote he states it to have been the "prevailing idea that it (effusion) is the uniform consequence of a wound of the intestines," whence many cases were abandoned as hopeless which might have recovered under suitable treatment, and Mr. Travers, therefore, held it to be very important in reference to the management of such accidents to establish that fæcal effusion was a comparatively rare event—a proposition which we think Dr. Gross has not shaken. Cases of, and experiments on, wounded intestine with prolapse returned without suture, have, we repeat, no bearing on the question at issue; and when we look to the numerous cases in which wounds of the intestines have been inflicted without fæcal effusion, together with the various experiments which have been performed respecting this point, we think there are good grounds for concluding that Mr. Travers's position comes much nearer to the truth than does that of Dr. Gross. Mr. Travers, we may here observe, terms Petit's statement, that fæces when effused may form a circumscribed depôt, a mere "conceit;" and avers that "in all cases which give the slightest countenance to this opinion a rupture had pre-existed, by which the gut had contracted an adhesion to the peritoneum." This is a point to which Dr. Gross does not refer; but the two following cases prove that effused fæces may be perfectly circumscribed. The first occurred in the practice of M. Jobert. A man was stabbed in the abdomen, and a portion of wounded intestine which presented at the wound of the parietes was sewed and returned; the patient died in thirty-eight hours, and on dissection another wound of the small intestine was discovered six feet from the stomach, whence had resulted an effusion of fæces, which was perfectly circumscribed on every side by the intestines, the mesentery, and false membranes. (Archiv. Gén. de Méd. 1837, p. 306.) M. Baudens records a case of gun-shot wound of the abdomen, in which the parietal wound was dilated, eight inches of small intestine included between two wounds of the bowel removed, and the extremities of the gut united by Lembert's suture: the patient died on the third day, and on dissection there was discovered a wound of the cæcum, with effusion of fæces, which was perfectly circumscribed and isolated by adhesions. The adjacent peritoneum was inflamed, which M. Baudens thinks was the cause of death, as adhesion of the reunited intestine had commenced. (Clinique de Plaies d'Armes à Feu, p. 335.) Both Mr. Travers and Dr. Gross agree that fæcal effusion into the abdomen is of necessity fatal. Were the fæces diffused, such must, doubtless, be the result; but Mr. Nourse's well-known case (Phil. Trans. 1776)—and others of the same kind might be quoted—is an example of confined fæces ultimately escaping by the external wound and sloughing of the abdominal parietes, and the case terminating favorably. This, it is true, has only occurred, so far, at least, as we are aware, when the fæces were circumscribed in the vicinity of the wound of the abdominal parietes; but it yet seems to be possible, in cases like those just cited from Jobert and Baudens, where effused fæces are circumscribed at a distance from the external wound, that they might, as has happened with effusions of blood similarly circumscribed, be discharged by suppuration making its way externally.

In the second chapter, on "Treatment of wounds of the intestine," Dr. Gross first considers "penetrating wounds of the abdomen unattended with protrusion of the intestines;" a section which is very judiciously written, with the exception, as we think, of one or two points on which we cannot coincide with the author.

When there is reason to suppose that fæces are effused within the abdomen, Dr. Gross recommends cutting down on the wounded bowel, removing the effused matter, and securing the wound of the bowel by suture. We certainly would approve of this practice if the occurrence of fæcal effusion could be diagnosed; but it is there the difficulty lies. Mr. Travers, Dr. Gross observes, with most surgeons, opposes searching for a wounded bowel, "on the grounds that the intestinal aperture retains its apposition with the parietal orifice; but," adds Dr. Gross, "he has adduced no experiments or facts of any sort in support of this conclusion, which is, besides, at variance with the existing state of our knowledge in relation to the subject." (p. 36.) We are here completely at issue with Dr. Gross. So far from failing to adduce facts in support of his position, Mr. Travers quotes numerous "instances of complicated intestinal wounds unattended by prolapse;" which, he says, "appear" to him "to authorize" his "conclusions." (p. 152.) Those cases surely are "facts," and important ones too, and we think, along with a host of others, abundantly establish that the wounded gut usually, to adopt Mr. Travers's words, "retains its apposition to the peritoneal wound;" a proposition which, though we think Mr. Travers states it too generally, as he does not admit, at least does not hint at, any exception to it, so far from being at variance with, is, on the contrary, consonant with the existing state of our knowledge. We do not, however, deny that cases may occur in which it would be proper to search for and secure the wounded intestine, though, indeed, we find it difficult to specify the circumstances which would justify such a proceeding. Dr. Gross, "for the sake of being more fully understood, supposes a case," that of a man, after a hearty meal, stabbed in the abdomen, "the bowel is pierced, or it may be nearly divided, and there is a copious discharge of fæcal matter, both externally and into the peritoneal cavity, as is evinced in the latter event by the excruciating pain, the gastric oppression, and the collapsed condition of the sufferer." (p. 34.) We need not stop to show that the symptoms here laid down as diagnostic of fæcal effusion are utterly insufficient; we have no doubt Dr. Gross, on reconsideration, would admit them to be so himself, but the question is, can better be supplied? Dr. Gross, though he subsequently quotes some of the cases contained in the work of M. Baudens, which we have already referred to, does not notice the statements and practice of that surgeon respecting this important subject. M. Baudens maintains, from the experience of, as he states, upwards of one hundred cases, that in penetrating gunshot wounds traversing the portion of the abdomen occupied by the intestinal canal, the latter is wounded in at least nine out of ten cases; and although no very alarming symptoms occur at first, fæcal effusion almost constantly exists, which will terminate fatally, unless the surgeon dilate the external wound, removes the effused matter, and sews the intestine. Larrey, it will be recollected, also considers fæcal effusion a very frequent consequence of gunshot wounds. M. Baudens, moreover, lays down certain signs whereby, he says, the fact of the intestine being wounded may be determined. According to him, the parietes of the intestine when it is torn by a gunshot wound lose their softness and pliancy, contract spasmodically, and acquire an almost cartilaginous hardness; the finger then should be passed through the external wound into the cavity of the abdomen, and, as the wounded bowel almost uniformly lies immediately behind the external wound, the foregoing condition of the intestine will generally be felt, and if so, M. Baudens says, we may certainly conclude that the intestine has been opened. If fæces escape externally, or soil the finger, of course no doubt can exist. And, furthermore, we may also conclude that the bowel is wounded, if gentle pressure on the abdomen expels bubbles of air through the wound of the abdominal parietes. If none of those symptoms exist we may infer that the intestine has escaped; but if any of them are present we should, he says, cut down on and sew the intestine. M. Baudens gives two cases in which he adopted this practice, in consequence of feeling the indurated condition of intestine above described, when he passed his finger into the wound. One of those cases we have already mentioned; in the other, after the external wound was dilated, the patient was desired to cough, on which a quantity of air, which had been effused into the abdomen, escaped, and the intestine protruded: a wound was discovered in the arch of the colon, which was secured by three points of Lembert's suture, and the patient recovered. M. Baudens, it is to be observed, confines his observations to gunshot wounds exclusively, and whatever may be the case after that description of injury, as the indurated condition of intestine he describes certainly does not exist after incised or punctured wounds, one of the diagnostic marks he mentions would in such cases be absent; nor, indeed, does it at all appear that M. Baudens considers fæcal effusion to be of frequent occurrence, except after gunshot wounds. However, one sign which he mentions, namely, tympanitis (which M. Jobert considers so important in rupture of the intestine without any external wound), would certainly seem to be of some value in any case; and if it be admissible to speak conjecturally on such a point, we would be inclined to say that tympanitis rapidly following a wound of the abdomen, especially if air could be expelled through the wound, would entitle us to rationally conclude that the intestine was open, and that fæcal effusion had either taken place or was to be apprehended, and certainly, if external issue of fæces were conjoined with or had preceded tympanitis, we would deem a surgeon justified in searching for the wounded bowel. The two cases, however, already quoted from MM. Jobert and Baudens show the uncertainty that attends diagnosis of wounds of the abdomen, but the complication that existed in those cases scarcely affects the point we are now considering.

The next section is on "Penetrating wounds of the abdomen, with simple protrusion of intestine, or omentum." There is, perhaps, no point on which surgeons are so unanimous as that a protruded and unwounded intestine should be replaced in the abdomen. The only writer we recollect who hints at any limitation to this rule is Delpech, who recommends us not to interfere if the gut adheres to the lips of the parietal wound, because adhesions limit inflammation, which might spread were they broken up; and experience has shown that a considerable portion of protruded intestine may heal over and continue to perform its functions. Dr. Gross says that to leave an intestine unreduced is a practice which must be "speedily followed by the death of the patient, or, what is scarcely less pitiable, an artificial anus." (p. 37.) It is, however, perfectly certain, as Delpech above incidentally states, that neither of those results must necessarily follow, as appears from several remarkable cases. Dr. Cochrane informs us that at St. Christopher's, in 1778, a negro stabbed himself above the navel; "an expert surgeon" found a considerable portion of bowel protruding, which he attempted to reduce after dilating the external wound; but, being foiled by the obstinacy of the patient, the "expert surgeon," at last, "got out of temper," when "the manager, knowing the worthlessness of the negro, locked him up, and, in a great measure, neglected him, thinking his recovery impossible." Some days subsequently Dr. Cochrane met the man walking to town, "supporting, in a coarse woollen blanket, the protruded intestines," which formed a mass about the size of a child's head; and, to the increase of the doctor's amazement, he found that the negro had been bathing in the sea. Ultimately, "the guts" became covered with a cicatrix, and the negro "could undergo any labour, and had no other inconvenience than supporting the tumour, which resembled the mamma of a woman." (Medical Commentaries, by A. Duncan, vol. x, pp. 276-8.) M. Lessiere records a case of protrusion of the omentum and stomach, complicated with a wound of the latter organ. The omentum was tied and cut away; and, as the patient was threatened with suffocation at every attempt to return the stomach, it was left unreduced. On the fourth day, a surgeon attempted to sew the wound of the stomach, to prevent the escape of the food; but the thread cut through the coats of the organ. In about two months the stomach had gradually and completely retracted within the abdomen, and the visceral and parietal wounds healed perfectly. (Mém. de l'Acad. de Chirurg., t. i, pp. 59-24.)

Though no doubt exists as to the propriety of reducing a simply protruded portion of bowel, there is some difference of opinion as to how protruded omentum should be dealt with. Dr. Gross very properly, as a general rule, recommends it to be returned, and deprecates the practice of Larrey, who, he says, "has advised us to let it alone." (p. 39.) But this is an inaccurate statement of Larrey's advice, who distinctly recommends the omentum to be returned, if seen before it has become considerably swollen, and, in that event, to dilate the external wound, if the omentum is thereby strangulated. We certainly consider those rules of practice more judicious than our author's recommendation, that protruded omentum "should *always* be carefully returned;" for our practice should vary according to the particular circumstances of the case.

In reference to the reduction of protruded intestine, Dr. Gross correctly observes, "it is all-important to know that it has actually slipped into its natural situation." (p. 38.) Arnaud and Garengeot long since warned surgeons not to return the bowel into the sheath of the rectus when the parietal wound involved that muscle; to this Dr. Gross does not allude, but he mentions another possible mishap which has been little noticed, viz. passing the intestine between the peritoneum and the abdominal muscles. As this is an event which may appear very little liable to happen, and as Dr. Gross refers to no case in which it occurred, it may be well to notice one recorded by Mr. Ellis. A man received a wound about three fourths of an inch long in the abdomen, from which nine inches of intestine protruded; with much difficulty, and after about forty minutes' perseverance, the gut was put out of sight, and in forty-eight hours the man died. On dissection, Mr. Ellis found a considerable portion of the bowel placed between the peritoneum and the abdominal muscles. (Lancet, 1834-5, vol. ii, p. 755.) A similar but not identical misadventure, not noticed by Dr. Gross, consists in pushing the intestine between the abdominal muscles, an example of which occurred in the practice of M. A. Berard. A portion of intestine prolapsed from a wound a little above Poupart's ligament, and was returned with apparent facility, but uniformly presented again externally after having been seemingly reduced. At length the gut remained up, and M. Berard, aware of the possibility of what had actually happened, passed his index-finger into the abdomen in order to assure himself that the intestine had been regularly replaced. On the fourth day after the receipt of the wound the patient died of peritonitis, which evidently commenced at and spread from the site of the wound, and on dissection a portion of intestine was found in a cavity between the external and internal oblique muscles. (Gazette des Hôpitaux de Paris, June 1842.) Those two cases show that such accidents may occur in the practice of surgeons of unquestionable skill and information. Mr. Ellis and M. Berard both ask whether the separation of the tissues into which the bowel was insinuated in their respective cases, was made during the attempts at reduction, or whether it had been in the first instance replaced within the abdomen, and had subsequently gotten into the situation in which it was found, and they both answer that the bowel was placed during the attempts at reduction in the positions above indicated, in which we think they are perfectly right. In both those cases reduction was extremly difficult, and perhaps, therefore, when such difficulty occurs there may be additional reason to suspect the possibility of the existence of a parietal pouch, and to take every possible precaution against passing the intestine into it.

"Penetrating wounds of the abdomen, with protrusion and injury of the intestines." After a judicious section on the therapeutic means to be adopted in those cases, Dr. Gross proceeds to discuss their treatment by different kinds of suture, but it seems more methodical to first consider the methods of treatment without any suture of the wound of the intestine itself.

When a wounded intestine protrudes, surgeons are by no means agreed as to what practice should be adopted. Among the older surgeons, Heister, Garengeot, De la Faye, Dionis, Sharp, Palfin and others, inculcated returning the intestine without suture when the wound is small; Le Dran and B. Bell, on the contrary, recommend the smallest orifice through which the contents of the gut could escape to be sewed up. Among modern surgeons, Mr. Travers, though he concludes from experiments that very small wounds may be safely returned, yet recommends the wound, however small, to be secured by suture, though M. Jobert represents him as attributing with Scarpa the most mischievous consequences to sutures, and utterly rejecting them. (Traité des Malad. Chirurg. du Canal Intest., t. i, p. 73.) Mr. Lawrence dispenses with the suture in a mere puncture, but recommends its employment if fæces could possibly escape through the aperture. Boyer considers the suture indispensable in wounds of the intestines exceeding four lines in length; Richerand rejects it in wounds not more than two or three lines long; Vidal de Cassis recommends its application when the wound is two lines long; and Jobert says we may safely return a wound three lines long, and, *à fortiori,* a puncture, even though a little fæces exude from it. Callisen, Richter, Marjolin, Begin, and Gibson say that the suture should not be applied in small wounds of the intestines, but none of them specify what extent of wound may be safely left to nature; and Mr. Gibson, as if mistrusting his own precept, during an operation for strangulated hernia, successfully imitated Cooper and Lawrence, by tying a ligature circularly round a small aperture in the intestine. Mr. Syme speaks doubtfully on this matter, but thinks it prudent to make a point of suture when the wound exceeds a mere puncture. Finally, the suture is altogether rejected by some, as, for example, Scarpa, who, however, admits that "a timorous surgeon," afraid "to commit the whole to nature," might "with impunity pass a ligature through the mesentery opposite the seat of the wound of the intestine," a proceeding which others, with J. Bell, more timorous still, replace by stitching the wound of the gut to that of the parietes to the abdomen.

Dr. Gross states that the method of simply returning a wounded intestine without suture was proposed by Scarpa. He considers it to be a plan even "more extraordinary and unaccountable" than that of J. Bell, and states that it, in common with J. Bell's method, is now universally condemned. The fact, however, is that this method is a very old one, was practised long previously to the time of Scarpa, and has frequently succeeded: that it is very easy, moreover, to account for its having been proposed, as the practice is a very legitimate inference from the ideas once generally held respecting the reparation of wounds of the intestine, and that so far from its being universally condemned, many modern surgeons recommend it, and we have some recorded examples of its having been adopted very recently. As to the method having originated with Scarpa, Tulpius (Obs. Med. lib. iii, cap. 20) and Hollerius (Obs. et Cons Curandi, p. 17), who are referred to by Mr. Travers, each record a case in which this mode of treatment succeeded perfectly, the wounds having healed thoroughly after artificial anus had existed for some time: Tulpius's case is the more interesting, as it is, we believe, the first example of a wounded intestine having been dissected long after union; the wound, we are told, was closed by a dense firm cicatrix. Ravaton's practice also was to return a wounded intestine without suture; he condemned the suture as not merely mischievous but murderous, and deemed it to be the essential point not to heal the outer wound till the fæces ceased to pass by it; with which view he sewed the upper portion only of the external wound, and kept a tent in its lower part; and he gives two cases which were thus treated and recovered perfectly. Several modern authors also favour, more or less, the method improperly termed Scarpa's. Delpech, in every case, Richerand, when the wound exceeds three lines in length, and Marjolin (Dict. en vol. 21, t. xvii, pp. 107 et seq.), in extensive transverse wounds and those with loss of substance, recommend that method, with the precaution of passing a thread through the mesentery. We find too, that in addition to the older cases, there are a few of very modern date, in which Scarpa's plan, so called, was adopted. One such case is mentioned in Scarpa's work on Hernia; and we may also refer to the following cases: A female lunatic wounded herself in the abdomen, and cut away seventeen inches of the small intestine, with a portion of the omentum, weighing one ounce and a quarter. One end of the gut had retracted within the abdomen, the other protruded, presenting an oblique ragged edge. Dr. Buttolph, deeming death inevitable, replaced the protruding portion of gut within the abdomen, and carefully stitched the external wound. No peritonitis supervened. Thirty-three days after the injury there was a scanty discharge of hard fæces, on the following day a copious alvine evacuation occurred, and all then went well. Two years subsequently the woman was in good health. (American Journ. of Med. Sc., by Hayes, 1835.) A man was gored in the abdomen by a bull, a portion of protruding small intestine was perforated by the animal's horn, and its contents issued freely from the wound; Dr. Mayberry returned the intestine into the abdomen without sewing it, and a fæcal fistula resulted, which, four months after the accident, occasionally gave exit to a very little liquid fæces. (Dub. Med. Press, 1842, p. 376.) Reybard also, in a case of wound involving about two thirds of the circumference of the colon, replaced the intestine unsewed, and retained it in the vicinity of the parietal wound by means of a thread in the mesentery; the patient recovered with an artificial anus. (Mém. sur le Trait. des Anus Artif. &c. p. 43.) Velpeau also having in an operation for strangulated hernia returned with impunity a portion of bowel in which three small orifices existed, ventured in another case to replace a wound of the intestine *two thirds* of an inch long, and the patient recovered. The last case of this kind which we shall mention is that of a man who having been stabbed in the abdomen, a wound six lines long was seen in a protruded portion of small intestine; the surgeons who first saw the patient retained the bowel between the lips of the external wound by a thread in the mesentery; the patient was then carried to La Charité, where M. Roux withdrew the thread, and returned the bowel into the abdomen without sewing it; death occurred on the second day, when fæces were found effused into the abdomen. (Gazette des Hôp. de Paris, 1835, p. 1.) Those cases show that Scarpa's practice is not universally repudiated, and in the two last cited we find it was adopted by surgeons of the highest reputation. We certainly, however, agree with Dr. Gross that it deserves to be rejected, and the last case well illustrates its danger, which might be further shown by several others: for example, in Cooper's folio work on 'Hernia' (pp. 32-34), two cases are given in which, during operations for crural hernia, the gut was either torn or cut in one, and perforated in the other, and in both inadvertently returned, the breach not being perceived at the moment; the patients both died with fæcal effusion into the abdomen.

Among the foregoing cases we have seen one in which a single extremity of a completely divided intestine protruded from the abdomen. The possibility, however, of this event has been doubted, and we do not find it noticed by Dr. Gross. John Bell sneers at B. Bell's recommendation, that in such a case we should search for the other end, and stitch the two together; saying, "as for intestines cut fairly across in all their circle, I believe the thing cannot happen, and that this, like all the rest, is a piece of mere guesswork: for, if I know anything about the way in which the viscera are disposed within the belly, it must happen that a sabre which cuts one piece of intestine fairly across, must have many others torn half through," and then he adds, we may "just as well let the poor man alone."

Roux also says (Nouv. Elémens de Méd. Opérat.) he does not know "that a case of an intestine completely divided by a wound has ever been observed." Such cases are, unquestionably, of very unfrequent occurrence, and are very difficult of explanation, except where, as in Dr. Buttolph's case, a maniac severs the protruded bowel; there are, however, some examples of the intestine having been completely divided by a simple stab. Garengeot gives the case of a soldier who was wounded in the lower part of the right epigastrium; the ileum was completely cut across, and one end of the gut protruded from the wound. The wound was judged mortal, "and the poor man was let alone," but he recovered with an artificial anus. (Traité des Opérations, t. 1.) The following case occurred in the practice of M. Berard. An assassin was carried to the hospital of St. Antoine, after having received in the left inguinal region a sabre wound 3½ inches long, from which about two feet of small intestine protruded, the bowel was completely divided, and one of its extremities only depended externally; M. Berard attempted in vain, by methodical traction on the mesentery, to discover the concealed end of the bowel. Matters were then left to themselves, and the man died in a few hours. On dissection the depending end of the gut was found to be the lower end, while the upper extremity lay close to the internal orifice of the wound of the abdominal parietes, and could have been easily reached by dilating the wound a little upwards and following the mesentery. (Gaz. des Hôpit. de Paris, 1832, p. 269.) Despite J. Bell's sarcasms, we cordially concur in the practice recommended by B. Bell in such cases. It may be right to add, that in M. Berard's case just mentioned, J. Bell's speculation was partly borne out, as several coils of the intestine were slightly notched—very slightly so, however; and so far from the bowel being "torn half through" in several situations, its cavity was scarcely laid open, except at the point where it was completely severed.

What is commonly termed John Bell's method, viz. stitching the gut to the external wound, should, Dr. Gross states, rather be termed the method of Palfin, as having originated with that surgeon. This is, however, a mistake. The method in question was, we believe, first proposed by De Blegny, who, having witnessed a wound of the ileum in which the gut adhered to the external wound, and the patient recovered with an artificial anus, instituted experiments on dogs, and finding that they recovered, though with artificial anus, after the intestinal canal had been opened and stitched to the external wound, proposed this mode of treating wounds of the intestine in the human subject. (Acta Eruditorum, 1682, t. i, p. 22.) The date of Palfin's Anat. Chirurg. is 1734 (not 1743, as Dr. Gross states), so that supposing there was any parity between De Blegny's and Palfin's methods, the former anticipated the latter by upwards of sixty years. But dates are here of no importance, for Palfin's method is entirely different from that proposed by De Blegny and adopted by J. Bell, as we shall presently see. Nor was this method forgotten between the days of De Blegny and J. Bell: Amyand, for example, surgeon to St. George's hospital, writing in 1736, says, "the readiest way to obtain a cure of a wounded or bursted gut, is to keep it in contact with the outer wound and the patient on a very low diet." (Phil. Trans. 1736.)

Respecting J. Bell's method (for so we shall term it for convenience sake), Dr. Gross says, "that it might occasionally be attended with success is not improbable" (p. 106), but that the verdict of the profession is entirely against it. Now the truth is that the method has frequently succeeded; B. Bell, for example, saw two cases of completely divided intestine, in which the extremities of the gut were stitched exactly opposite each other to the lips of the external wound, and recovery ensued without the formation of an artificial anus; and Hennen mentions two cases of oblique wound of the colon, upwards of an inch long, in each of which he secured the gut by a single stitch to the parietes of the abdomen, and both patients recovered in a few days. (Military Surgery, p. 416.) As to the verdict of the profession having been pronounced against this mode of treatment, we really are not quite certain but that the authorities might nearly, if not quite, preponderate in its favour; Dr. Gross himself cites Professors S. Cooper, Gibson, and Syme as giving it their sanction, and Hennen, we have seen, adopted it,—but to this point we shall return.

Dr. Gross, while condemning J. Bell's method, resolved to test its value by experiment, though Dr. Smith and Mr. Travers had, he says, already exposed its insufficiency. Mr. Travers, however, has not published any experiment conducted according to J. Bell's method. Dr. Gross refers to his Experiment R (p. 116) as being such, but it is, on the contrary, the very reverse, for in it the lips of the wound of the bowel were brought into contact by a single point of suture inserted opposite the mesentery, the gut was returned, and the external wound sewed. It is right to add that Mr. Travers by no means gives this experiment as a trial of J. Bell's plan; he adduces it to show that the interrupted suture favours fæcal effusion more than no suture at all, as each stitch, he maintains, becomes the extremity of an aperture the area of which is determined by the distance of the stitches. Dr. Smith's experiments, indeed, purport to be trials of J. Bell's method, but they are not; for he distinctly says that the intestinal wound "was *secured by one stitch, and* fastened to the wound of the parietes." Dr. Gross's three experiments, intended to test the value of J. Bell's method, not only have no resemblance to it, but are the very reverse of it; they are simply repetitions of Mr. Travers's experiment just noticed, one stitch having been taken *in the wound of the gut* and the external wound *closed.* Consequently, neither Dr. Gross's experiments nor those of Dr. Smith were trials of J. Bell's plan, and Mr. Travers's neither was, nor was intended to be so; yet Dr. Gross says, "thus, in seven experiments, all conducted, there is reason to believe, with the requisite care and skill, not a single one had a favorable termination." (p. 108.) The truth is, J. Bell utterly deprecated any suture of the wound of the intestine; his recommendation was to secure the bowel by a single stitch to the outer wound, which was to remain open to give free exit to the fæces. Dr. Smith's experiments, above noticed, were close imitations of Palfin's plan, the only difference being that Palfin, after making a single stitch in the wound of the gut, simply brought the extremities of the thread through the external wound, instead of fastening the bowel to the external wound. We are not aware of any case in which Palfin's method has been adopted in the human subject.

Dr. Gross, we have seen, considers Palfin's, J. Bell's, and Scarpa's methods as "extraordinary and unaccountable." Palfin, he states, "entertained the singular notion that the divided ends" (of the intestine) "never united with each other, but that the cure was effected solely by the adhesions which they formed to the surrounding parts." (p. 105.) Now this gives the explanation of the puzzle, and fully accounts for the proposal, and, till very recently, the nearly general adoption of the modes of treatment we have been considering. Palfin's notion, respecting the mode of reparation of a wounded intestine, instead of being singular, is as old as the days of Hippocrates:—"Si quod intestinorum graciliorum discinditur non coalescit" is a dictum which still passes current. Surgeons have always been guided in their practice by their views respecting the process whereby a wounded intestine was repaired, and, as they almost universally assumed that the intestine could only heal by adhesion to the adjacent peritoneal surfaces, they not illogically rejected sutures as tending to excite inflammation; and, accordingly, Morand, Ravaton, Sharp, Scarpa, Sabatier, Delpech, Leveillé, J. Bell, Richerand, Begin, Reybard, Roux, Patissier, more or less completely reject the suture, while others, with Boyer, merely adopt it as a temporary resource against fæcal effusion. The opponents of the suture also deprecated it on grounds which we find admitted to be valid by some of its warmest advocates. Thus Mr. Travers admits that the issue of fæces externally in wounds of the intestines "relieves the patient from local and constitutional disturbance" (pp. 153-55); that, in such cases, "the symptoms are generally less imminent than of those in which the external communication does not exist" (p. 138); and, finally, that "the passage of the matters by the wound does not in any degree impede the subsequent efforts of nature to restore the canal." (p. 157.) These are, in fact, the very arguments of those who deprecated the suture, they held that dispensing with it was the safer practice, and not likely to be followed by artificial anus. Within the last few years, indeed, sutures, so applied as to bring the serous surfaces of the intestine into contact, have come into favour; but most authorities hold with A. Berard (Dict. de Méd., 2d ed., vol. xvii, pp. 57 et seq.) that, in wounds of the intestine above a few lines long, Scarpa's or J. Bell's method would be preferable to any of the old sutures. Larrey, indeed, is the only continental writer we recollect, who, until very recently, held that the structures of the intestinal canal could adhere mutually by the action of their own vessels, and who accordingly adopted the suture with the intention of producing such union.

Before considering Dr. Gross's experiments respecting the process of reparation, we shall advert to his experiments respecting the phenomena presented by a wounded bowel, and the action of a ligature on the intestine. Dr. Gross's results, respecting the former point, are generally similar to those of Mr. Travers, and MM. Jobert and Reybard, but they are more precise, being estimated by measurement, and differ, in one respect, as regards the behaviour of longitudinal wounds. Reybard states that a longitudinal wound of the bowel becomes elongated; a wound two inches long, for example, becoming two and a half inches long; Travers's statement is to the same general effect. Dr. Gross found—1. That a longitudinal incision $2\frac{1}{2}$ lines long contracted to $1\frac{3}{4}$ line, and was completely closed by the everted mucous membrane. 2. A similar wound, 4 lines long, contracted, in a few seconds, to 3 lines by $1\frac{1}{2}$ in width; and the protrusion of the mucous membrane left no perceptible opening. 3. An oblique cut, 7 lines long, contracted to 5 by $2\frac{1}{2}$ in width. 4. A transverse wound, $2\frac{1}{2}$ lines long, was immediately reduced to a rounded opening 2 lines in diameter. 5. A transverse incision half an inch long became an oval opening 4 lines long by $2\frac{1}{2}$ wide.

Dr. Gross repeated several times Mr. Travers's celebrated experiment, of firmly tying a small ligature round the intestine of a dog, and with the same general result as Mr. Travers; a few points, however, deserve notice. Mr. Travers and M. Jobert both state that a ligature, firmly applied, divides all the coats of the intestine, save the serous; and, according to M. Jobert, the latter is also ruptured at several points by an unwaxed thread, which should not therefore be employed in sewing an intestine. Dr. Gross found that, even when a small ligature is very tightly constricted, the cellular and muscular coats are but partially divided. Neither Travers nor Jobert say anything as to the time the ligature takes to cut into the cavity of the intestine. Dr. Gross, in one case, found it through more than half the circumference of the bowel on the third day; in another experiment it passed completely into the cavity of the gut in $3\frac{1}{2}$ days: in both those experiments the intestine was found constricted, the opposed mucous surfaces, lying in contact in one, the canal being partially pervious in the other. The escape of a ligature, however, is extremely variable; in another experiment it was found imbedded in coagulable lymph at the point where it had been applied on the eleventh day. We observe, Dr. Marshall Hall states (Lancet, 1837-8, p. 73), that, in performing this experiment, it is necessary to the restoration of the continuity of the canal that the animal shall have fasted twenty-four hours, and that a portion of intestine near the stomach shall be operated on. It does not appear that either Mr. Travers or Dr. Gross adopted the former precaution. Mr. Travers, indeed, operated on the duodenum, but Dr. Gross incidentally says that the experiment succeeds perfectly on the large intestine. (p. 26.)

Though Dr. Gross's results respecting the process of reparation of a wounded intestine by suture have developed nothing absolutely new, they elucidate, indeed we think decide, a much disputed point. Mr. Travers states that the contiguous mucous surfaces when approximated by suture agglutinate, but that this union is never permanent, being destroyed by the action of the longitudinal fibres of the bowel when the ligatures loosen; and this, he states, is the reason why the thread invariably passes into the intestinal canal. But, even if this retraction could be prevented, Mr. Travers thinks the mucous surfaces are incapable of uniting either directly or by granulation, and that, consequently, the breach is never obliterated internally. (Op. cit., pp. 130-2.) Jobert, on the contrary, affirms that the mucous membrane is reproduced at the line of division, and expressly states that Travers is wrong on this point. (Op. cit., p. 78.) M. Reybard, we believe, was the next to examine the action of the suture; he did not, like Travers, observe any separation of the lips of the wound after their primary agglutination by interposed lymph, on the contrary, he found them remain closely approximated; but he agreed with Travers that the edges of the divided mucous membrane never united with each other, the interspace between them remaining occupied by the originally effused lymph, which becomes an organized cicatrix depressed below the level of the mucous membrane at each border, but presenting a prominent ridge along the centre where it is thickest, and adhering to the serous and muscular coats of the intestine only, as is easily shown after a couple of days' maceration, when it can, he says, be detached in its entire extent; and this cicatrix, he thinks,

M. Jobert probably mistook for regenerated mucous membrane. (Journ. Complém. des Sc. Méd., 1830.) Dr. Gross, who does not notice M. Reybard's experiments, observed that the early cohesion between the lips of the wound very commonly, though not uniformly, persisted when the suture loosened; he also found that the adhesion of the mucous membrane, though achieved more slowly than that of the other tunics, is ultimately complete, and may occur in one of two ways. Usually the lymph effused between its divided edges is gradually absorbed, until the edges themselves come into contact, "and, after a period varying from a few weeks to as many months," coalesce directly; sometimes, however, but not frequently, the breach of the mucous surface is healed by granulation. The appearances observed in Dr. Gross's experiments are detailed with such precision as must, we think, satisfy every reader of their accuracy, and that they definitively settle in the affirmative the question of the regeneration of the mucous membrane of the intestine. It is satisfactory, however, that Dr. Gross's conclusions are strengthened by experiments performed, or at least published, almost concurrently with his own. M. Reybard's opinion has been completely altered by new researches, and he now admits that the mucous coat can cicatrize completely like the other tunics (Journ. de Méd. de Lyon, October 1843, and L'Expérience, December 1843); and M. Petrequin has also fully ascertained that the mucous tunic is regenerated, the only perceptible alteration being that the villous surface is thinner than natural, and that a slight depression exists at the seat of reparation. (Anatom. Méd. Chirurg.)

"Treatment of wounds of the intestine by different kinds of suture." Though it is fully established that a wounded intestine may be completely united by suture, it remains to be determined what is the probability of union occurring in wounds of various extent and direction, and what kind of suture is preferable. Dr. Gross first treats of the "continued" suture, which, he says, is synonymous with the glover's suture, and considers Bertrandi's suture, or the "*suture à points passés*" as different from the continued suture. (p. 101.) It is scarcely necessary to say that those two sutures are merely two varieties of the continued suture.

The experiments which have been performed with the glover's suture are very numerous. Shipton cut away two inches of the ileum of a dog, and united the extremities with the glover's suture; on killing the dog several weeks after, the ends of the bowel communicated through a kind of cyst formed by adhesions among the surrounding parts. Dr. Wallis mentions that a horse having been staked and the stomach wounded, a farrier emptied the stomach, sewed the wound, and "then thrust the maw back again into the body, and stitched up the wound in the rim of the belly;" in a few weeks the animal worked as usual. (Phil. Trans. 1695). Brunner sewed a wound one inch and a half long in the small intestine of a dog with the glover's suture; the dog soon recovered, but it is difficult to say what he would not have recovered from, as, subsequently, milk was injected into his thorax, his femoral artery was tied, his spleen was extirpated, his pancreas was cut away, and, finally, he was compelled to swallow a scruple of opium, and all without serious consequences, as he made his escape three months after the last attempt on his life. (Exper. Nova circa Pancreas Præf. pp. 6-7). According to Louis, Mœbius having failed in effecting Ramdohr's method of invagination in dogs, sewed the ends of the bowel together, but the animals died with fæcal effusion into the abdomen. (Mém. de l'Acad. Roy. de Chirurg. t. iii.) Dr. Thomson in two dogs, Sir A., then Mr. Cooper, in one, applied the interrupted and glover's suture conjointly to wounds of the small intestine an inch and a half long; two of the dogs survived, a third died with fæcal effusion. (Cooper on Hernia, fol. pt. i.). Mr. Travers performed experiments also with the glover's suture, but deemed an account of them superfluous. M. Reybard, from his earlier experiments in 1830, already alluded to, concluded that the glover's suture was the best mode of treating a wounded intestine, an opinion in which he is confirmed by, and which M. Petrequin has adopted from, their more recent researches.

Dr. Gross performed 17 experiments to ascertain the effects of the glover's suture. In 2 the bowel was wounded transversely, and in 1 completely divided. In 12 the wound was longitudinal, varying from half an inch to 6 inches, and in 3 oblique. All the animals recovered, but 3 of them "were killed too soon after the operation to render it at all certain that they would have recovered from the effects of it." (p. 64.) We shall give the summary of the particulars of those experiments in Dr. Gross's words.

"In 8 the needle was carried through the whole thickness of the bowel, and in 5 the everted mucous membrane was pared off on a level with the surrounding surface; in 8 the suture was introduced through the fibrous lamella, or between the muscular and mucous coats, and in 1 through all the layers of the tube, except the peritoneal. It is worthy of remark that the caliber of the tube was not sensibly diminished by the operation in any of the experiments.

"Of those three methods, that of introducing the suture through the cellulo-fibrous lamella is the least objectionable, as it enables us to bring the serous surfaces into more accurate apposition. Where the needle is conveyed through all the tunics there must necessarily be some degree of puckering, whereby the mucous lining will be forced between the lip of the wound, if not beyond the level of the peritoneal coat. By such an arrangement the adhesive process would be retarded, and if the stitches were to lose their hold, or if the bowel should not become glued to the neighbouring parts, fæcal effusion might occur." (pp. 64-5.)

Dr. Gross, and MM. Reybard and Petrequin all dwell on the importance of taking the stitches of the glover's suture very close to each other, and drawing them firmly in order to ensure union. The French writers also recommend, as essential to ensure the passage of the suture into the bowel, to take the first stitch from within outwards, and to make a very large knot on, or even attach a small dossil of lint to, the extremity of the thread.

Dr. Gross considers that "the results of his experiments are eminently favorable to the use of the continued suture" (p. 64), and certainly, so far as they go, they are. In one dissection only was the breach found partially blocked by adhesion of an adjacent peritoneal surface; but here a wound six inches long had been inflicted, upwards of three inches of which was closed by the transparent mesentery. The earliest date at which the suture was found to have completely passed into the intestine was the 35th day; in other instances it was found partially adherent on the 22d, 28th, and 31st days, and its separation does not appear to have been at all influenced either by the everted mucous membrane having been pared off, or by the needle having been carried through the fibrous lamella of the bowel, a point which we notice for the following reason. Since Dr. Gross's work was published, M. Moreau-Boutard has proposed to sew wounds of the intestines by excising the everted mucous membrane, and bringing the peritoneal coat and the exposed submucous tissue into contact, and he effects the latter object (as we infer, for in the abstract it is not very distinctly stated) by passing the sutures through the submucous cellular tissue. M. Jobert, in his Report to the Academy of Medicine, on M. Moreau-Boutard's Memoir, objects to this proposal that it would often be physically impossible in the human subject, that it incurs the risk of the intestine mortifying, from being deprived of nutrition, and, finally, that the suture cut short on the external surface of the bowel must almost infallibly be detached into the cavity of the peritoneum; for, according to M. Jobert, unless a suture traverses all the coats of the intestine, it is quite uncertain whether it will pass into the cavity of the bowel, and if it does not it either remains in the cicatrix, where it may excite inflammation and suppuration, or becomes detached into the peritoneum. It is, therefore, only when all the tunics are perforated that the suture can, he says, be safely cut short, and if the mucous coat is not pierced, the thread should, he maintains, depend from the external wound, to admit of its escaping when detached; M. Jobert adds, that a surgeon, who was ignorant of this mechanism of the elimination of sutures, had the mortification to see a patient on whom he had performed suture of the intestine die of peritonitis, excited by a suture falling into the peritoneal cavity. (Bulletin de l'Acad. Roy. de Méd. Sept. 1845, pp. 1036-40.) M. Jobert does not give any particulars of the case just referred to, nor does he cite any experiments of his own in evidence of this point, neither does he say that the sutures had not passed into the bowel in any of M. Moreau-Boutard's experiments, but simply alleges that his method is attended with great danger of their not doing so. M. Reybard, however, expressly says that if sutures do not embrace the entire thickness of the bowel they should not be cut close, as they will not then pass into the cavity of the intestine, but cause abscesses on its external surface, as he observed in experiments on dogs in which the sutures did not penetrate the mucous membrane. (Journ. Compl. 1830, t. xxxvii, p. 21.) But, on the other hand, there are experiments which lead to the directly contrary conclusion. Dr. Thomson found that the suture passed into the bowel when the peritoneal coat *alone* was included in the suture; we have seen that in Dr. Gross's experiments the separation of the suture was not influenced by the coats of the bowel it was passed through, and we may add, that in those experiments in which it was carried through the submucous tissue, and the animal was killed before its complete separation, it had made considerable progress towards being thrown off into the cavity of the bowel. Coupling, however, M. Jobert's statement with M. Reybard's positive experiments, we must conclude that this point requires re-examination, and that it would be scarcely prudent in practice to cut short a suture which included a portion only of the tissues of the bowel. M. Jobert's other objections to M. Moreau-Boutard's method are merely speculative, and seem to us to be unfounded, but the method itself, probably, does not possess any peculiar advantage.

Dr. Gross concludes the section on the glover's suture by detailing six cases: that of Mr. Travers, of Lisbon, two from Glandorpius, one from the Dict. des Sc. Méd., and two from Larrey; of those cases two only were fatal. We are aware of but a few additional cases in which this suture has been employed. Guy de Chauliac mentions a successful case in which he sewed an intestine "vulnerata secundem longum et latum, cum sutura pelliparioum." (Ars Chirurgica, 1546, p. 336.) Fallopius, we think, records a similar case, and Garengeot mentions a patient who died the third day after suture of the intestine was practised by Guerin. (Traité des Opèrations, t. i. p. 191.) The most remarkable case, however, of the application of the glover's suture on record is one published by M. Reybard. A man, aged twenty-eight, laboured under a carcinomatous tumour of the sigmoid flexure of the colon, and M. Reybard having diagnosed the affection, cut into the abdomen, cut away three inches of the intestine affected with the disease, tied the arteries of the mesocolon, and united the extremities of the divided bowel with the glover's suture, the suture was cut short, the intestine replaced in the abdomen, and the wound of the parietes sewed with three stitches. On the 10th day a copious evacuation was passed per anum, and on the 38th day the patient was pronounced well. After the lapse of six months, symptoms of cancerous disease of the intestine again set in, and the patient died about twelve months after the operation. No examination of the body could be obtained. This case was communicated by M. Reybard to the Academy of Medicine of Paris, and M. Jobert, in a report thereon, stated that M. Reybard had performed experiments, before a committee of the Academy, on seven dogs, but in not a single instance was union of the bowel obtained; fæcal effusion occurred in all, in some diffused through the abdomen, in others circumscribed by adhesions between the intestines. The committee consequently concluded that the glover's suture cannot be relied on in the treatment of wounds of the intestine, and we certainly think that however favorable many experiments may seem to be to it, it is far inferior to suture with inversion of the serous surface of the bowel. (Ann. de la Chirurg. Fr. et Etr. Aug. 1844. p. 493.)

We may pass briefly over Dr. Gross's experiments on the interrupted suture. In fourteen experiments death occurred once only (and that after complete division of the bowel), though in some the stitches were four and five lines apart, and in no instance was the caliber of the intestine diminished. In ten experiments the sutures were cut short, and in all they either passed or were in progress of passing into the cavity of the bowel. We are surprised Dr. Gross fails to remark the great rapidity, as compared to the glover's suture, with which single stitches are detached. The latest period at which the sutures were found adherent was the 17th day, and in two experiments they were detached by the 7th and 11th days respectively. Dr. Gross concludes this, like the preceding section, by quoting a number of cases in which the interrupted suture was employed in the human subject, which, he observes, "taken in connexion with the experiments just detailed, exhibit an array of success highly favorable to this method of treatment." (p. 82.) Those cases are seven in number, five successful, and two fatal; the two latter are extracted from Cooper's folio work on Hernia; three of the former occurred in America, and two are taken from the 'Edinburgh Medical Surgical Journal' (vol. xii, p. 27), and the 'Medico-Chirurgical Review' (vol. xx, p. 182). We may just add that in the latter journal (vol. vi, p. 557) there is another case mentioned, in which Dr. Washbourn united several small wounds of the intestine by the interrupted suture with success, and in the Journ. der Practisch. Heilkunde, Feb. 1825, is a remarkable case, in which Dr. Fuchsius, having diagnosed the existence of intussusception of the intestine, cut into the abdomen, drew out the invaginated bowel, and, being unable to free it, cut into the gut and disengaged the invaginated portion, which was two feet in length, united the wound by sutures, and the patient recovered in fourteen days.

Dr. Gross next (certainly very unmethodically) introduces a section on the "Method of Ramdohr," the history of which is very accurately given, and Dr. Gross adds to the cases of its application in the human subject that are generally known, one from the American Journal of Medical Science (vol. x, p. 42.) In this case the lower extremity of the intestine is supposed to have been passed into the upper extremity, as what seemed to be the lower end was separated from the mesentery by the knife which had inflicted the wound; the invagination was maintained by three points of suture, two other wounds of the small intestine were secured by the continued suture, and a single stitch was taken in a wound of the colon; the patient was well by the 19th day.

The short section on Le Dran's suture requires no comment, and but little need be said respecting that on the "Method of Bertrandi, or la suture à points passés." Dr. Gross says that, excepting Boyer, he knows of no modern advocates of this suture: it is, however, recommended by Sanson, Roux, Patissier, Lombard, Richerand, and others, but by the last writer in long wounds only. Dr. Gross says we have no cases of its application in the human subject, and it is remarkable that we have very few, considering how long this suture was favorably regarded by many continental surgeons. Lombard, however, mentions one case, (Clinique des Plaies Recentes, p. 197): in 1778 an English soldier was landed at Gravelines with a sabre wound in the hypogastric region, through which the ileum protruded, and was wounded to the extent of more than a finger's breadth. Lombard sewed the wound with Bertrandi's suture; all went well till the ninth day, when Lombard lost sight of the case, and did not learn its issue. The following is also an example of the use of Bertrandi's suture, as the mucous surfaces were directly confronted. M. Judrin, operating on a femoral hernia nine days strangulated, found a perforation two lines in diameter in the intestine; finding that two stitches à points passés suffered fæces still to exude, M. Judrin took two other stitches at right angles with the former, the gut was returned, and the patient recovered in a few weeks. (Gazette Méd. de Paris, April, 1840, p. 250.)

Dr. Gross, in his account of the "Method of the Four Masters," only alludes to a single experiment on this method performed by Sir A. Cooper. Louis informs us that Duverger tried the method on dogs previous to his well-known application of it in the human subject. Louis himself tried it on two dogs, and they recovered. (Mém. de l'Acad. Royale de Chirurg. t. iii.) Vogel tells us that Thomas Brayn, a veterinary surgeon, in 1704, opened the abdomen of an ox affected with obstinate constipation, cut away a mortified portion of intestine, and sewed the ends of the bowel over a tube of wood; the cylinder was soon voided per anum and the animal lived several years. (Sandifort, Thesaur. Disputation, etc., vol. ii, p. 130.) Dr. Watson proposed this method as a new one so lately as 1790, and having excised a portion of the bowel of a large dog, sewed the divided extremities over a cylinder of isinglass. The animal, "recovering perfectly, seemed not to suffer any inconvenience from having had his gut shortened four or five inches." (Medical Communications, vol. ii, p. 111.) But, though successful in those experiments, Duverger's case is, we believe, the only authentic example of its having succeeded in the human subject, or, indeed, rather the solitary instance of its having been so applied, for it seems uncertain whether Jamerius, Roger, Theoderic, William of Salicetus, or the Four Masters, really ever practised this method, or merely recommended it theoretically.

The next section, entitled "Method of Palfin, Bell, and Scarpa," we have already disposed of. The following one is devoted to the "Method of Jobert."

Dr. Gross tried Jobert's method of invagination on two dogs, both died, one on the third, the other on the seventh day; on dissecting the latter animal, he found that the lower extremity of the intestine had been introduced into the upper, "into which it projected (firmly united to its inner surface) in the form of a mammillated protuberance, six inches in length, tapering at its free extremity, and perfectly closed" (p. 118); the intestine, consequently, was completely obstructed, and was distended with gas and fæces to at least five times more than its natural size above the obstruction. Indeed this method has proved very unfortunate in the hands of every experimenter whose account we happen to have met with, excepting always M. Jobert himself. M. Begin tried it on three dogs, they all died, one from fæcal effusion, two from peritonitis. (Recueil des Mém. de Méd. de Chir. et de Pharm. Milit., t. xxii, 1827, pp. 60 et seq.) Reybard, in fifteen experiments on dogs, passed the lower into the upper end of the gut five times, and fæcal effusion uniformly occurred: in the ten others, in which the upper extremity of the bowel was invaginated, fæcal effusion also occurred in two; three of the remaining dogs died within a month, and the five that survived that period remained emaciated, were subject to frequent vomiting, and when killed for examination, it was found that the bowel had contracted almost to obliteration at the seat of invagination, above which, it was greatly dilated, and correspondingly contracted below. (Jour. Complém. des Sc. Méd., t. xxxviii, pp. 337 et seq.) M. Petrequin's experiments with this method were also so unfortunate, that he unreservedly condemns it. Dr. Gross also rejects this plan because of its difficulty, of the violence done to the parts in executing it, and of the uncertainty of its result; and we must entirely concur in this condemnation, were it for no other reason than the impossibility of distinguishing the lower extremity of the intestine with certainty; but that distinction, according to Jobert himself, is so essential to success, that he proposes to adopt Louis' method of discriminating the upper extremity of the intestine, a proceeding which, we trust, needs no refutation. Dr. Gross, after adverting to J. Cloquet's case, in which this method promised to succeed when the case was reported, but the ultimate issue of which we do not know, mentions another, and a fatal case, in which Professor Berard, relying on the alleged sign that the lower orifice of a divided intestine is more contracted than the upper, invaginated the lower extremity of the bowel into the upper one. There is, however, a circumstance of some interest in this case which Dr. Gross omits to notice; the intestine was furnished with valvulæ conniventes nearly to the cæcum, and it was this circumstance that induced M. Berard to attempt invagination instead of allowing an artificial anus to form; for as he supposed that the small intestine was divided high up, because of the existence of valvulæ conniventes at the site of the wound, he feared that an artificial anus would have been followed by death from defective nutrition, had the patient survived the immediate consequences of the wound. There are two other cases not mentioned by our author. One is mentioned by M. Jobert. (Archiv. Gén. de Méd. 1824, t. iv, p. 74.) A woman wounded herself in the throat and abdomen; the divided intestine was invaginated, and she died in fifteen hours from the effect of the wound of the throat, but soft filaments of lymph were found interposed between the portions of the bowel. The second occurred in M. Jobert's practice at the Hospital St. Louis. A man, aged 70, cut away a portion of his intestine during the night, and the injury was not discovered till next day, when, in addition to the wound with loss of intestine, it was found that the bowel was completely divided in a second situation; this latter wound was united by an invagination retained by five points of simple suture; the extremities of the portion of the canal which had suffered excision were kept in contact with the abdominal wound, in hopes of establishing an artificial anus. In thirteen hours the patient died, yet adhesion had made such progress that the invaginated serous surfaces remained glued together after the sutures were removed. (Annales de la Chirurg. Fr. et Etrang. Dec. 1842, p. 437.) M. Laborie, who records the foregoing case, adds, that M. Boulen has successfully treated *two* cases of complete division of the small intestine, by M. Jobert's method of invagination, and that M. Jobert himself had lately performed the same operation with perfect success on a patient of M. Beaumes, in whom the intestine had become gangrened in a crural hernia. No particulars are, however, given respecting the three last cases, and, indeed, the precise mode of invagination practised is not clearly stated as regards the two former either, so that the four last, at all events, of those five cases may be, and we suspect probably are, examples of Lembert's rather than of Jobert's method. Dr. Gross does not appear to have tried experimentally M. Jobert's suture for partial wounds of the intestine; M. Reybard objects to this method, that the kind of valve which results from it would be dangerous in transverse wounds, and that simply collecting and twisting the sutures together and bringing them through the parietal wound, without knotting them, is insufficient to keep the lips of the wound of the intestine inverted and in contact, as he experimentally ascertained in dogs. This objection we find to some extent confirmed by a case published in the 'Annales de la Chirurgie.' (loc. sup. cit.) M. Jobert united a wound in the small intestine three centimetres long, with three sutures inserted according to his method, and instead of knotting the threads, twisted them together, and allowed them to depend from the external wound; the patient died next day from peritonitis and a vast effusion of blood in the pelvis; the wound of the intestine had almost all united, but was patulous at one angle.

Dr. Gross performed twenty-three experiments with Lembert's suture, but he neither deduces any conclusions from them, nor gives any analysis of the results. In nine of the experiments the bowel was completely divided, and four of those animals died, two with fæcal effusion. The results of the experiments on partial wounds of the bowel with this suture are very favorable, one dog only perished; those experiments also show the rapidity with which single points of suture pass into the bowel; in one experiment they had disappeared on the 12th day, and were never found at a much later date. The caliber of the intestine does not appear to have been in any instance diminished even when the wound was two and a half inches long, nor do the inverted lips of the wound seem to have permanently projected into the bowel; in one dissection, on the 17th day after a wound two and a half inches long had been united by eight points of suture, it is merely stated that "the villous margins of the wound were a good deal more elevated than common, but it was evident that they were everywhere continuous with each other." (p. 140.)*

We pass the "Method of Denans" and its modification by Baudens, nor shall we stop to notice a somewhat analogous, but even more objectionable proceeding proposed by Spillmans, an account of which may be found in the Memoir of M. Begin, which we have already referred to. As regards the "Method of Reybard," we shall only observe that M. Reybard has long since renounced his own method, and strongly pointed out the disadvantages and dangers attendant on it. Dr. Gross says he is not aware that this method has been employed on the human subject; but it has, and with success, by its inventor, who, notwithstanding, we repeat, unreservedly condemns it. The case in which M. Reybard applied his own method, is, we may observe, recorded in M. Reybard's 'Memoir on Artificial Anus,' &c., a work to which Dr. Gross himself refers.

Dr. Gross finally considers the methods of Amussat, Thomson, Choisy, and Beclard, which are all mere speculative proposals founded on Mr. Traver's celebrated experiment, in which the continuity of the intestine was restored after it had been circularly constricted with a ligature, and severally consist in so constricting the extremities of a divided intestine after they have been invaginated, either simply, as Beclard proposed, or over a tubular apparatus of more or less fanciful construction, as devised by others. On these speculations we need not dwell; but we may here notice the result of the only case we are aware of, in which a human intestine was completely included in a ligature; the case is mentioned by Reybard. (Mém. sur le Trait. des Anus Artif., etc. pp. 86 et seq.) A child, aged between six and seven, was wounded in the abdomen, the colon protruded, but was not wounded, and the surgeon, if such he is to be called, who saw the child, tied a silk ligature round the bowel and replaced it, the result of this strange proceeding was, that symptoms of strangulation came on, and continued until the ligature cut through the coats of the intestine, and an artificial anus resulted. It is well to know that such a proceeding is not necessarily fatal in the human subject.

Of the preceding methods of suture, Dr. Gross prefers the glover's

* Dr. Gross quotes several cases in which Lembert's suture was employed. We are not aware of any other cases besides those quoted by Dr. Gross, and we give the references to them, as so doing may prove convenient to some of our readers. 1. M. Jobert, Recovery (Lawrence, Treatise on Ruptures, p. 306; Velpeau, Médecine Opérat., t. iv, p. 143). 2. Dieffenbach, Complete division of the intestine, recovery from operation, death several weeks after from internal strangulation (Lawrence, ut supra, p. 362; British and Foreign Medical Review, vol. iii, p. 517). 3. Jobert, Recovery (Archiv. Gén. de Méd., March, 1837). 4. Baudens, Recovery (Op. sup. cit., p. 336). 5. Liegard (Velpeau, Méd. Opérat., t. iv, p. 143). 6 and 7. Jobert, Fatal (Lawrence, Op. sup. cit.), 8. Baudens, Fatal (Op. sup. cit.)

suture and that of Lembert, without awarding a preference to either, except, indeed, the whole cylinder of the intestine were divided, in which case he would prefer the glover's suture, "especially in young subjects, in whom the canal is very narrow, or in persons in whom the bowel is overloaded with fæcal matter at the moment of the injury," as "in a case of this kind the inverted edges might occasion serious obstruction, from the manner in which they project into the interior of the canal." (p. 157.) For our own part, we would certainly prefer Lembert's suture, or that of Gely, in every case where we deemed a suture applicable. We confess, however, that we do not think it is yet satisfactorily determined what is the most advisable course to pursue when an intestine is completely divided by a recently-inflicted wound, and we are by no means satisfied but that the safest course, in such an event, is to establish an artificial anus, taking care so to dispose the extremities of the intestine as to favour attempts to subsequently heal the wound; to discuss this point suitably would, however, at present carry us too far. Dr. Gross, we may here observe, states in the next chapter (for he is eminently unmethodical in his arrangement and discursive in his observations) that he would deem it advisable "in extensive longitudinal or oblique wounds, to excise the affected part, and treat the case like one in which the tube is completely divided in the first instance . . . . especially where the opening is more than two inches in length." (p. 171.) In this opinion we can by no means coincide; Dr. Gross's reasons for adopting it are—that extensive wounds take a long time to heal, that the canal may become permanently contracted, that adhesion in them is seldom complete, that numerous sutures are a source of irritation, and that mischief must result from the protracted manipulation necessary to apply them. We do not dispute the force of those reasons, but most assuredly they apply *a fortiori*, one and all, to complete division of the intestine, and, *if* valid, the necessary inference from them is, that extensive wounds of the intestine, and still more complete division of it, should be treated, not by suture, but by the establishment of an artificial anus.

It has been objected, and not without some reason, to Lembert's suture, that the wound is not securely closed unless the points of suture are tolerably close, and that the danger of inflammation increases with their number. M. Gely, to obviate this inconvenience, has proposed a modification of Lembert's suture, which purports to combine the advantages of so thoroughly closing the wound as to obviate all danger of either immediate or secondary effusion; of presenting such an arrangement of the suture that neither the knot nor any portion of it is visible on the peritoneal surface, and that it must certainly pass into the intestine; and finally, of being easily executed and applicable in every case. This suture may be practised with one or with two needles. When two needles are employed, which is the preferable mode, one of them is inserted a line or so behind, and external to one of the extremities of the wound, is carried parallel to the wound for two or three lines in the cavity of the intestine, and is then brought out on the peritoneal surface again: precisely the same is done with the opposite needle; the threads are then crossed, the left needle is now introduced into the puncture through which the right needle has just passed, and a stitch similar to that first made is taken with it, and a like stitch, in like manner, is made with the right needle. As many stitches are thus made, parallel to each side of the wound, as its extent may require, and it then only remains to tie the threads and tighten the stitches sufficiently; this is accomplished by pulling the threads at each point where they cross the wound with a dissecting forceps, at the same time inverting the lips of the wound, which soon become so perfectly coapted that the thread is completely concealed between them; finally, the extremities of the thread are knotted and cut close, and the knot is as effectually concealed as the rest of the suture. The needles should be fine, but a little larger than the thread, to permit the latter to run with freedom; when the needles are crossed, it is not indispensable to pass them exactly into the orifice which the opposite one has traversed, and it both facilitates and hastens the operation to tighten and knot the threads each time they are crossed: in complete division of the intestine this is indeed indispensable. This mode of suture may, perhaps, seem difficult when thus described, but it is really easily performed, as we have ascertained by trial; the essential point is to make the corresponding stitches at the opposite sides of the wound of equal length, as otherwise the wound is puckered. It is unnecessary to describe the manipulation when a single needle is employed. M. Gely states, that when the completely divided intestine of a dog is united by his method the resulting valve produces nearly complete temporary obstruction; but the valve gradually diminishes, and perhaps it may finally disappear, as we find, that in one of his experiments, five and a half months after complete division of the intestine, the valve had disappeared round half the circumference of the bowel, and in the other half resembled one of the valvulæ conniventes somewhat thickened. The suture applied in this way is detached very slowly: in one experiment it was found adhering in the wound two months after having been applied. M. Gely's experiments on animals with this suture are scarcely sufficiently numerous, but so far as they go are favorable to the method. M. Gely has, also, twice applied his suture in the human subject. The first case was that of a sailor, who received a wound in the left lumbar region, through which about a yard of small intestine protruded; the prolapsed bowel presented two small wounds exactly opposite to each other, having been transfixed by the knife; each wound was secured according to the method already described; the intestine was reduced, and the patient recovered without having presented any very serious symptoms. In the second case, M. Gely inflicted a small wound on the intestine during an operation for strangulated hernia; he secured the wound by a stitch of his suture, returned the intestine, and the patient recovered.

We may here mention that M. Hip. Nunciati of Naples, has proposed a *spiral* suture, by which the serous surfaces of the lips of the wound of the intestine are inverted and brought into contact; it may be that the account we have seen of this method is imperfect, but it seems to us quite analogous to the modification of Lembert's suture already proposed by Dupuytren. M. Nunciati's suture, is performed with a single thread, which is carried along the wound alternately from left to right, and from right to left, and then, by pulling the extremities of the thread, one at each angle of the wound, the lips of the wound are inverted and brought into close contact. M. Nunciati is said to have treated three cases successfully in this way, but we are not acquainted with their particulars. (Bulletin de l'Acad. Royale de Méd., Sept. 1845, p. 1041.)

None of the old sutures are applicable in wounds with loss of substance, involving a portion only of the circumference of the intestine; but M. Gely maintains that his method is perfectly suited for such a case. When so applied, the intestine must of course be flexed on itself, and the more so, the greater the loss of substance. The resulting curvature of the bowel, M. Gely maintains, from the results of experiments on animals, does not cause any obstruction or other inconvenience, even when the inflected portions of the bowel are placed parallel to each other. Again, if two orifices, with loss of substance, should exist in the intestine, M. Gely proposes that they should be brought in contact by means of his suture, which is as easily applied to two orifices as on the two margins of one aperture. But the few observations we have to make on this head may conveniently find place in a brief notice of Dr. Gross's third chapter, entitled "Of the treatment of wounds of the intestine by ligature and excision."

This chapter relates entirely to the conduct which should be pursued when a portion of intestine is gangrened in a strangulated hernia. Dr. Gross first alludes to the case of a minute orifice in the intestine, and notices the practice of encircling it with a ligature, which we have already referred to; but we may here allude to a case which shows that a ligature thus applied does not uniformly make its way into the cavity of the bowel. A man received a sabre wound in the abdomen, two and a half yards of the bowel protruded, and at one point, near the mesentery, presented a wound about the size of that commonly inflicted in venesection; it was uncertain whether this wound penetrated all the coats of the intestine; but Dr. Kothe, to render matters secure, pinched up the wound in a forceps and included it in a small circular silk ligature, which he cut close to the knot. All went well till the sixth day, when peritonitis set in, and the patient died on the ninth day. On dissection, the ligature was found lying loose on the surface of the jejunum, and though the intestines were removed from the body, no trace of the wound could be found, though it was supposed to have pierced the bowel, as the patient had passed bloody stools. The failure of the ligature to cut into the bowel, in this case, may have arisen from its not having been applied sufficiently tight, but this, of course, is matter of conjecture. (Lond. Med. Gaz. 1827-8, vol i, p. 807, and Rust's Magazine, 1828.) As to the practice to be adopted when a considerable portion of intestine is gangrened, Dr. Gross cites several cases to show that patients may survive the loss of even several feet of the intestinal canal. Among these, he alludes to Mr. Needham's case in the 'Philosophical Transactions,' as being that of a boy who had fifty-seven inches of his bowel "cut off by a cart;" but that is not exactly the fact; the case, indeed, is a curious one,—the pressure of a cart passing over the abdomen forced a large portion of the intestine, with part of the mesentery, out of the anus. Every attempt at reduction failed, as the bowel was constantly again protruded by retching, and on the third day, as the parts appeared to be mortified, Mr. Needham cut them away. The greatest loss of intestine we recollect, where the patient survived, occurred in an operation for hernia by Arnaud, who cut away "more than seven feet" of gangrened bowel (A Dissertation on Ruptures, pp. 341 et seq.); but all this has really nothing to do with the matter at issue; if an intestine is gangrened it is already lost, and the only question is, shall the surgeon suffer an artificial anus to form, or endeavour to restore the continuity of the canal.

In considering this question, Dr. Gross leaves out of sight that an opportunity of interfering in the latter way really very seldom occurs, for the bowel has commonly become adherent above the mortified part, and we believe no surgeon disputes the rule that such adhesions are not to be disturbed. But suppose the bowel is not adherent, which Dr. Gross seems to assume is the usual condition of things, he leans to the opinion that, "when there is much inflammation beyond the sphacelated parts, it would probably be wrong to pursue any other treatment" than to suffer an artificial anus to form, but "if, on the other hand, the tube is nearly or quite sound, I should not hesitate to excise the mortified structures, and to approximate the ends by the suture." (p. 173.) M. Gely thinks that his suture may be applicable in cases of gangrened intestine, when a portion only of the cylinder is involved; but if an entire zone of the tube is destroyed, he prefers the establishment of an artificial anus, but, at the same time, proposes an expedient which seems to us extremely plausible and well worthy of consideration, as probably presenting the advantages of the suture without its dangers: this expedient is, to unite by his suture the extremities of the intestine, not completely, but in a third or one half, of their circumference only; obstruction to the course of the fæces would be thus avoided, and the bowel favorably placed for healing the artificial anus. M. Gely further asks, might not a notch be cut in the free border of each of the portions of intestine thus united. This would be, in fact, anticipating the action of Dupuytren's enterotome on the *eperon.* M. Gely has found this answer perfectly on dogs, but speaks with fitting reserve as to its application in the human subject.

Dr. Gross's fourth and last chapter, is on "Artificial Anus," and in it the pathology and treatment of the affection are fully and clearly considered. This chapter cannot be expected to present very much novelty, and but a few points call for observation.

Dessault, Noel, and others, have succeeded in curing artificial anus by obliterating the prominence of the *eperon* by pressure, applied directly on it by tents. Dupuytren also tried, but was compelled to abandon more forcible pressure on the *eperon.* Since Dr. Gross wrote, Mr. Trant communicated to the Surgical Society of Ireland an extremely ingenious instrument, which seems admirably suited for accomplishing everything that can be achieved by compression on the *eperon;* this well-devised contrivance presents the great advantage, that while very considerable pressure can be exerted on the *eperon,* there is not the least danger of the force applied tearing the adhesions of the bowel to the abdominal parietes, because the bowel is pressed from within outwards against the parietes of the abdomen, with a force equal to that exerted on the *eperon.* This instrument perfectly succeeded in Mr. Trant's hands in one case of artificial anus, and we have little doubt that future experience will confirm its utility. (Dublin Med. Press, vol. xiii, p. 305.)*

Dr. Gross mentions the unsuccessful attempts of Bruns, Liotard, and Blandin to obliterate an artificial anus by approximating with sutures its edges previously rendered raw by the knife or by caustic. Cooper, in his folio work on Hernia, mentions two other similarly unsuccessful attempts. Dr. Gross also notices the successful autoplastic operations of Mr. Collier and M. Blandin, an unsuccessful attempt of the same kind by Velpeau,

* As an adequate idea of this ingenious instrument could scarcely be conveyed by a mere verbal and also the method of the last surgeon, which consists in uniting by suture the raw edges of the opening, and facilitating the approximation of the parts by making a semi-elliptical incision on each side, and about an inch from the preternatural orifice. There are some other very interesting cases in which artificial anus has been latterly cured by autoplasty, either alone or combined with other methods, and which show what perseverance may accomplish in remedying this lamentable infirmity. Dr. Gross quotes Reybard's treatise on artificial anus, but does not advert to his cases, further than to say that two of his patients rapidly recovered after the application of his enterotome, whereas of his three cases one only was cured by the enterotome, a second remained unimproved, and in the third, which recovered after a series of autoplastic operations, the enterotome was not used at all. This last case is the remarkable one already description, we copy, by permission of the proprietor, from the 'Dublin Medical Press' the accompanying figures, which both represent the instrument itself, and also exhibit it as applied in the treatment of artificial anus.

Fig. 1.

Fig. 2.

Fig. 3.

Fig. 1 represents the position of the eperon after the third application of the instrument with it *in situ* : A. The upper or ventricular portion of intestine. B. The anal or lower portion of intestine. C. The mesentery, by its retraction towards the spine, assists the propeller E in removing the eperon F from the preternatural opening. D. The instrument introduced into the cavity of the intestine, with the propeller E pressing back the eperon F towards the spine, whilst the expanded wings GG on the inside, and the shield H on the outside, act as a forceps in retaining the intervening parts in close apposition, so as to prevent any separation of the anterior surface of the intestine from the parietes of the abdomen, whilst the propeller is pressing back the eperon towards the spine.

Fig. 2 represents the instrument with its wings expanding, and the propeller pushed forward, as in the cavity of the intestine, during application. A. The body. BB. The right and left wings. CC. The screws by which the respective wings are moved. D. The propeller. E. The concave extremity of propeller. F. The end of propeller by which the concave extremity is pressed against the eperon. G. Screw for fixing propeller. H. Shield which is moveable. I. Screw for fixing shield.

Fig. 3. J. The key by which the different screws are turned.

alluded to, in which a ligature was tied round the colon. The artificial anus occupied that bowel, and was upwards of an inch in diameter. A large flap of skin was dissected from the abdomen and applied over the opening, where it perfectly adhered, except at one point, where a fæcal fistula about four lines in extent formed. The edges of this fistula were freely pared and united by suture, but union was prevented by the issue of fæces. M. Reybard was now about to abandon the patient, when he observed that the skin of the abdomen was thrown into large transverse folds when the trunk and thigh were mutually flexed: he then pared the edges of the fistula again, applied no sutures or adhesive plaster, but kept them in contact solely by making the patient sit with the trunk flexed forward, while the thigh was maintained flexed on the abdomen by a bandage passing under the ham and round the shoulders and loins. This position was observed for one month, when a very minute orifice only remained, which gradually healed under applications of nitrate of silver, and the patient finally and completely recovered. M. Lisfranc was less fortunate in an apparently more favorable case. In operating on an umbilical hernia he cut away about fourteen inches of gangrened intestine, an artificial anus resulted, and seven months after the operation he applied Dupuytren's enterotome, (the only example we recollect of its being employed in an umbilical hernia, with the exception of a case of M. Robert's*), and four months subsequently it was applied a second time; the fæces now resumed their course through the rectum, and a mere fistula remained at the umbilicus. After several months, the edges of the fistula were pared and united by the twisted suture, but adhesion failed from erysipelas: after three months an autoplastic flap was applied, but erysipelas again prevented union; eight months subsequently the twisted suture was again applied, but union again failed, though no erysipelas set in. (Gazette Méd. de Paris, 1843, p. 543.) M. Blandin, in a child who was wasting from an artificial anus high in the small intestine, after in vain trying pressure on the *eperon,* with Dessault's tent, and a special apparatus contrived for the purpose, applied the enterotome, after which the orifice contracted considerably, its edges were now pared, and the twisted suture applied, with the result of very slightly diminishing the opening; three months subsequently the edges of the fistula were again refreshed and approximated by means of two "*tampons articulés*" of Dupuytren, without using any suture; the apparatus was kept on for ten days, when union was complete, and remained permanent. (Bulletin de l'Acad. Roy. de Méd., Nov. 1844, t. x, p. 110.) In the following case M. Jobert resorted to a contrivance different from any of the foregoing. The patient had an artificial anus in the left groin, the result of strangulated hernia, which had existed nearly two years. Before he came under M. Jobert's care the enterotome had been applied several times without any amendment, and ineffectual

* In M. Robert's case the enterotome was applied within three weeks after the operation for strangulated hernia had been performed, as the patient was sinking rapidly from defective nutrition, and notwithstanding the very disadvantageous circumstance of so very early an application of the enterotome the case did well, and a slight fæcal fistula only remained. M. Voillemier, in a case in which the patient was similarly wasting away, succeeded in supporting the strength of the patient by nutritious enemata, until a sufficient period had elapsed from the performance of the operation for strangulated hernia, to allow of the enterotome being applied without incurring more than the ordinary risk consequent on the use of that instrument. The effects of nutritious enemata are, however, so very capricious, if we may use that phrase, that their beneficial influence cannot be calculated on with certainty under similar circumstances.

attempts had been made, after touching the borders of the orifice with caustic, to unite them by the pressure of a padded forceps, and subsequently by the twisted suture; repeated applications of the cautery had also proved fruitless. Under these circumstances, M. Jobert endeavoured to remedy the infirmity by applying an autoplastic flap, but hemorrhage came on, the summit of the flap became gangrened, and the residue contracted towards its base. After a suitable lapse of time, the actual cautery and nitrate of silver were repeatedly applied during a month, and produced some slight diminution of the aperture. Finally, M. Jobert performed the following operation: a portion of skin was dissected away at each side of, but at some little distance from, the artificial anus, and the raw surfaces were then brought into contact in front of the preternatural opening, and secured by six points of twisted suture. The parts thus united formed a kind of bridge in front of the artificial anus, with a re-entrant angle above and below at their point of contact. A little fæcal matter oozed from the lower angle on the second day, and from the upper angle on the third day, but the parts united in front of the artificial anus; the oozing of fæcal matter from beneath them gradually lessened, and after some months the cure was perfect. (Bulletin de l'Acad., etc. Sept. 1845, p. 1030.) Dr. Gross does not notice a particular mode of applying the actual cautery in artificial anus, recommended by Dieffenbach, which consists in destroying the edge of the bowel where it joins the integument, and also a considerable portion of the mucous surface within the orifice, without acting on the skin itself; in this way Dieffenbach closed an artificial anus, resulting from a lance wound, after an autoplastic flap had failed, (Kleiner's Repertorium, Nov. 1835,) and Dr. Fingerhuth healed two intestinal fistulæ consequent on abdominal abscesses caused by blows. (Wochenschrift fur die Gesammte, Heilkunde, No. 6, 1836.) Dr. Gross just mentions a very extraordinary case in which M. Roux endeavoured to remedy an intestino-vaginal fistula, but he does not mention the particulars, which are, we believe, without precedent. M. Roux opened the abdomen, divided the intestine in two places, immediately above and below its adhesion to the vagina, as he thought, and united the extremities by three points of suture, according to Lembert's method; the operation lasted an hour and a quarter, and the patient died in thirty-eight hours. On dissection it was found that the ileum had been divided about two inches and a half above the artificial anus, but instead of dividing the small intestine below that point, the operator had severed the sigmoid flexure of the colon, and sewed the small intestine to the lower extremity of the *upper* portion of the colon. (Recueil de Mém. de Chirurg. et de Pharm. Milit., t. xii, p. 213.)

We had hoped that Dr. Gross would have considered the question of the comparative curability of artificial anus arising from a wound and from strangulated hernia; we think that the greater difficulty of remedying the former has been assumed on far from sufficient grounds, and it is obvious how much this assumption must tend to influence the surgeon in the practice he adopts in certain cases of wounds of the intestine. But this point we cannot pursue further at present.

The length and minuteness of our examination of Dr. Gross's work sufficiently shows that we deem it an important one. The subject of wounds of the intestine required examination, and indeed we must say that, in our opinion, it still requires it. Dr. Gross has made a valuable contribution to this branch of surgery, but the field of inquiry he has selected is far from being exhausted. Dr. Gross's experiments constitute the most valuable part of his work, and we look on them as the most important body of facts of the kind yet published. Dr. Gross evidently possesses a high degree of experimental skill, and he as evidently is endowed with the rare and valuable faculty of accurate observation: nothing can be clearer than his account of the results of his experiments. We take our leave of Dr. Gross with thanks for the information, and even more for the materials for reflection he has afforded to us.

In the foregoing brief observations on artificial anus, the various methods of treating that infirmity have been alluded to, without entering into the details of the practical application of any of them. We think it may be useful to append a summary account of the methods recommended by Dieffenbach, in the 62d chapter, of the first volume of his work on 'Operative Surgery,' as his experience is derived from the treatment of a very considerable number of cases.

Dieffenbach states that he has found the application of pressure on the ridge intervening between the two extremities of the bowel, of the greatest advantage in the treatment of many cases of artificial anus. He applies it by means of

"A crescentic ivory crutch of the thickness of a quill, the staff of which passes through the pad of a gum-elastic truss, and is prevented from falling through by screwing on a small plate. The crutch is so placed upon the partition that one horn passes into each intestinal opening, the staff is passed through the pad, the small plate screwed on, the truss applied and fastened; and, lastly, the long staff projecting through the pad is pressed backward by a strap which runs over the small plate, and is fastened by a buckle. The pad lies immediately on the skin as in a common truss, and is protected by laying beneath it strips of dressing. The bandage is removed several times daily, in order to clear away the excrements, and, when the parts are cleaned, the crutch and truss are reapplied. Gradually the strap is to be buckled a hole tighter, in order more strongly to depress the partition." (Dieffenbach, p. 705.)

While this instrument is being worn, an attempt should be made to restore the function of the lower portion of the intestinal canal, which may have been partially or completely suspended, perhaps for years, and Dieffenbach says enemata of ale and porter are remarkably efficacious in fulfilling this indication. This treatment to be conducted safely, must be conducted slowly, for a hasty increase of pressure produces pain and inflammation, and obliges the treatment to be suspended for some days. If we thus succeed in obliterating the ridge, and bringing the orifices of the two extremities of the intestine opposite to each other, so that their posterior walls shall form a continuous surface, it still remains to close the external opening, and this Dieffenbach effects by the application of the cautery and of the running suture, as described in the following extracts from his work.

"*Application of the cautery.* The bandage is removed, the parts cleaned and dried, a roll of charpie is placed in the opening of the gut for its protection, and the extreme strip of intestine, just where it is united with the integument, is destroyed by a small bean-shaped iron, the narrower rounded ridge being applied. The iron is carried round slowly, a second is taken, and again carried round, so that a burnt ring two lines in breadth surround, the abnormal anus. The part is covered by a narrow roll of charpie, and then the bandage is reapplied without the crutch. After the separation of the slough a circular granulation springs up, the ends of the intestine sink still deeper, and the excrements pass off more freely by the natural channel. After a fortnight a surface of at least an inch of the skin around the opening is to be cauterized, and its inner edge also slightly so." (Dieffenbach, p. 706.)

Lint is applied, first a mild, then an irritating ointment is used, the patient well nourished, and an enema of beer given daily. By the process of cicatrization the opening is soon converted into a small fistula, which another cauterization and the pressure of a soft truss-pad suffices to close.

"*Application of the running suture.* The running suture is necessary after treating a very large preternatural anus in the way just described by the crutch or cautery, when the external loss of substance is very great, and the cautery has not produced the concentric cicatrix so efficient in closing the opening. A strong curved sewing-needle, armed with four or six threads of waxed silk, is carried around the opening, a third or half an inch from its edge. The needle is passed in and out, and again in and out, always being again passed into the same place where it was brought out, until the opening is completely surrounded. Six such punctures are generally necessary to encircle the opening completely. Both ends of the threads hang out from the first puncture, and are tied together with a loop upon a small roll of plaster laid below them, and thus the opening is either completely closed, or only narrowed according to the degree of tension. The threads should never be tied together tightly, because they would then cut through in a few days, as they lie beneath the skin, and only surround soft parts. As they become loose they are tied somewhat tighter, and this is repeated until the parts are cut through. Contraction of the opening is sometimes very considerable after the first suture, still a repetition after the cicatrization which succeeds, and another cauterization of the edges, is necessary for its perfect closure." (Dieffenbach, p. 708.)

When artificial anus results from a penetrating wound of the abdomen, from gangrene of a portion only of the circumference of the intestine, or from an abscess in the parietes of the abdomen opening both externally, and into the bowel, there is often great difficulty in closing the aperture, especially when the small intestine is involved. In these cases the prospect of effecting a cure is most favorable when the disease has existed for a considerable period, and all the parts connected with the preternatural opening (particularly the adhesions with the intestine) have become relaxed and extended; here, of course, the crutch is not applied as there is no ridge to be obliterated, and the cure must be effected by the actual cautery, the running suture, and autoplasty; singly or successively practised, according to the circumstances of the case. The following are Dieffenbach's observations respecting the application of those methods in this class of cases:

"A small hook-shaped iron red hot is placed upon the edge bordered with the mucous membrane, and this edge is destroyed; then the point of the hook is passed in through the opening, and some lines' breadth of the internal circumference is burnt. A roll of charpie, the middle of which is tied together with thread, is introduced for the protection of the neighbouring parts, and held fast by the thread. The opening is covered with charpie and plaster, and when granulation begins to show itself around, the wound must be gradually drawn together by strips of plaster. Afterwards the cauterization is repeated, especially around the external edge and border, and if the opening does not speedily contract, the running suture is applied around it, between the intestine and integument. There is more necessity for transplantation of skin in this than in any other form of preternatural anus, particularly when hard cicatrices surround the opening, as these render replacement impossible, and other means are ineffectual. In some cases I have perfectly cured with the cautery alone even when excision of the edges and suture, lateral incisions and transplantation of skin, either as a bridge or in flaps, have failed. One of the most difficult cases of preternatural anus occurred in a Polish officer, who, in battle, was wounded in the abdomen by the lance of a Cossack, the transverse colon being injured. After many fruitless attempts to cure, after transplantation of skin had proved unsuccessful, the application of the cautery succeeded, the destruction of the mucous membrane around the edge and on the internal circumference contributing most essentially to the cure." (p 713.)

Though Dieffenbach considers pressure on the ridge by means of his crutch the best and safest mode of proceeding when applicable, viz. when the partition lies at some depth, and the ends of the intestine form a right or an obtuse angle with each other: he considers that Dupuytren's enterotome may be indispensable when the ends of the intestine lie parallel to each other, like the barrels of a double-barrelled gun, or when the partition is tense and hard, and reaches to, or extends beyond, the external opening. Nothing requiring notice is said respecting the application of this instrument. We may, however, mention that, in Dieffenbach's opinion, none of the numerous modifications of Dupuytren's enterotome have increased the utility of the instrument, while many of them have impaired it; this latter observation particularly applies to all the modifications intended to remove a circular or oval portion of the partition, a proceeding which, however apparently advantageous, experience has shown to be prejudicial, because the concentric contraction consequent on the cicatrization of the circular aperture renders the opening much smaller than when Dupuytren's enterotome has been applied; but even his instrument may cause such great contraction as to render the disease incurable. Dieffenbach objects to Dupuytren's practice of opening fistulæ connected with an artificial anus, and removing any hard irregular cicatrices surrounding it, because loss of substance is the result, and new cicatrices form; he says, he has found it much more advantageous to dilate the fistulæ with sponge tents, and soften the cicatrices with poultices and embrocations.

Artificial anus, connected with an unreduced hernia, is a rare affection, but Dieffenbach appears to have met with several cases of it. The treatment he adopts is modified by the circumstances of the case. 1. The cure of the preternatural anus may be undertaken *without attempting to cure the hernia;* when the hernia is very large and adherent in several places, when one wall only of the intestine is perforated, when the intestine is not contracted, and the inguinal canal is so large that after closure of the abnormal anus, the excrement can pass freely through the protruded portion of the gut.

After the patient has been prepared for a long time by horizontal posture, spare diet, poultices, and beer enemata containing carbonic acid, cauterization of the opening is performed with the red-hot iron.

"A roll of charpie is first passed into the opening, and then its lip-like border of mucous membrane is destroyed with a small conical iron. Then a large iron, of the form of a piece of money, is taken and applied flat, so that the opening is under its middle. When the opening, for example, is of the size of an English silver twopenny piece, the round cautery which is applied should equal in circumference that of a shilling. The cauterization of the circumference must be slight, so that only a layer of skin is destroyed of the thickness of strong letter paper. When the operation is finished, the roll of charpie is removed from the canal of the intestine, and the whole of the parts are covered with some handfuls of soft cotton and a compress, over which a broad suspensory or T bandage is applied. The bandage is as frequently renewed as it is soiled, the patient kept on low diet, and a clyster administered daily at least. The granulations which spring up after the separation of the slough are to be protected from the excrements by dressing, afterwards a stimulating ointment is used, assisted by caustic, and any small opening which remains is closed by the running suture and repetition of the actual cautery. This method is best adapted for large scrotal herniæ. When there are several small openings, they are treated in the same manner, but when it can be determined that some of the openings are in the lower part of the intestine, or that the smallest portion of excrement passes through them, they are to be first closed, as their cure is more easy." (pp. 723-5.)

*Cure of the preternatural anus of a hernia, and of the latter,* can only be affected when the scrotal hernia is moderately large, when it contains only a simple loop of intestine, and when the ring is small. Only a strong young subject is fit for the operation.

The condition here is as follows. The contents of the intestine pass from the abdomen into the protruded gut, and then escape through the preternatural anus. The gut at the situation of the opening is large, the other part, particularly when it passes through the ring, is contracted, and when this state has existed long, it is so narrow that only a thick catheter will pass. The intestine frequently lies free in the ring like a reducible hernia, but sometimes adhesions have formed. When the intestinal opening communicates with that in the skin, the skin, sac, and intestine are all adherent to each other.

*Treatment when the hernia is moveable.* When the loop of intestine is only adherent at the situation of the preternatural anus, and the lower part of this loop is still in some degree wide and extensible, we must endeavour to dilate the rings by division with the knife without opening the sac. Then by gentle manipulation the upper part of the protrusion is gradually returned. This is possible without the use of the knife, if the rings are not very small. Before the wound has become cicatrized, the cautery is to be applied as before directed, and as the opening is diminished the intestine is pushed farther back into the abdomen. When the opening is perfectly closed, the intestine is replaced as far as possible. The portion of intestine which adheres to the integument is drawn outwards as a diverticulum, afterwards the cellular connexions become lengthened, and the hernia is prevented from reappearing by a truss.

*Treatment of preternatural anus in a small adherent hernia* consists—1, in destruction (verödung) or removal of the adherent loop of intestine; 2, in forming a common preternatural anus; 3, in healing the latter. We shall follow our author's description of this operation, as he says he has thus exactly detailed the state and treatment of a man 28 years of age, who, in a year, was cured perfectly of a scrotal hernia of the size of a fist, and of a preternatural anus which opened into it.

"The operation commences with division of the scrotal integument, and opening of the hernial sac, just as in the operation for hernia. The opening being carried to two thirds of the length of the tumour, the spot is reached where the loop of intestine passes through the ring. Without separating the adhesions at the ring, their union with the neck of the sac is to be loosened. When this is completed, the intestine is cut through close before the ring, first dividing the part in which the anal opening exists, and then, at the same height, the part which passes into the abdominal cavity. If the two intestinal extremities are not closely adherent to each other, their sides turned towards each other are to be united at their edge by a thread passing between their serous surfaces. The separated perforated loop, with contracted canal and hypertrophied walls, is loosened and removed from the hernial sac, and also any degenerated portion of the sac and scrotal integument.

"After the bleeding has ceased the cavity is filled with loose charpie, strips of plaster laid over it, and the whole covered by charpie, a compress, and T bandage.

"The bandage is removed as often as it is soiled, free evacuation of the excrement attended to, and after some weeks, pressing backwards of the intestinal extremities into the abdomen is effected by balls of charpie laid against their openings. The condition is then the same as if the loop of intestine had been lost by strangulated hernia, and a common preternatural anus had been formed. After some months, when the hernial sac is obliterated, when there is a free passage to the intestinal extremities, when the adhesions have become extensible, then the preternatural anus is to be treated exactly in the manner before described, by the application of the ivory crutch and elastic truss. If the inferior extremity be considerably contracted, it is to be gradually distended by tents; but if the crutch can be first brought to bear upon one of its walls, its gradual dilatation will be thus readily effected. Lastly, when the two extremities have been pressed deeply backwards into the abdomen, so that they lie with their openings towards each other, the excrements begin to pass by the anus, and the cure approaches. Closure of the external opening in the scrotum here offers little difficulty, as the intestinal extremities lie at a considerable distance from it, and no integument has been lost. The actual cautery is here seldom necessary, as the use of caustic will close any remaining fistula." (pp. 726-7.)

"In case the separated adherent loop of intestine should be left, in order to avoid an extensive wound, it would be advisable to destroy (veröden) its canal, by drawing a thick cotton wick through it, and, if necessary, this may be covered with an irritating ointment." (p. 728.)

The transplantation of an autoplastic flap is only required in the treatment of artificial anus, when the skin surrounding the orifice is hard, inextensible, and altered in structure; if it be soft, pliant, and extensible, the opening, even if large, may be closed with the running suture. If a ridge exist, it must, of course, be first removed, either by pressure or by the enterotome. It is unnecessary to describe the details of the operation; but Dieffenbach insists that the transplanted flap shall not completely obstruct the exit of the fæces in the first instance. His earlier operations sometimes failed from neglecting this precaution, and he, therefore, fastens the flap by suture in two thirds of its circumference only, the anterior border being left free to allow the fæces to escape. When the operation is completed, water is injected under the flap, and then charpie introduced beneath it, and the whole supported by strips of adhesive plaster, the free edge of the flap, however, being left uncovered. The chief point in the after treatment is to endeavour to prevent the passage of fæces for as long a period as possible, which is sought to be effected by perfect quiet, dry food in small quantity, small doses of opium, and small enemata of castor oil and camomile. If the dressings, on careful examination, do not appear soiled, they are not disturbed for three or four days; if soiled, they must be immediately renewed. The sutures should be removed about the eighth day. When the attached portion of the flap has completely united, the issue of fæces should not be opposed by strong pressure, but merely impeded by charpie, and endeavours made to heal the opening by granulation, by the application of resinous ointments, &c.; generally the opening gradually contracts to a mere fistula, which, by proper management, finally heals. If, however, the flap unites imperfectly, it is very apt to contract towards its base; which must, as far as possible, be counteracted by the judicious application of pressure, and keeping it on the stretch by adhesive strips of plaster, while granulation of its edges is at the same time promoted by resinous ointment. Fomentations with camomile sometimes appear to be extremely useful.

The formation of *a bridge-like flap* is preferable when the skin on, at least, one side of the opening is healthy and extensible—the orifice is circumscribed by two long concave incisions, vertical, transverse, or oblique, according to the existence and position of cicatrices, and the included skin is smoothly dissected away. An incision is now made parallel to one of the wounded edges. The strip of skin thus isolated must be one third wider than the opening. Its inner border is fixed with hooked forceps, and it is dissected from the subjacent parts in the interspace between the two incisions, and when thus completely free, except at its two extremities, it is drawn over the opening and attached to the opposite edge. The after treatment is the same as when a pediculated flap is transplanted.

We have space for but little comment on the foregoing summary of Dieffenbach's practice. We regard simple pressure on the ridge with great apprehension, because of the absence of a *point d'appui*, on the wall of the intestine, where it is attached to parietes of the abdomen, whence the risk is incurred of the adhesions in this situation being torn. But Mr. Trant's instrument (which, as we happen to know, Dieffenbach has seen) is free from this objection; for it supplies the *point d'appui*, and, consequently, there is no danger of the bowel being torn from its attachment. We cannot pretend to deny that the running suture may often be useful, and sometimes indispensable; but we are strongly inclined to the opinion that Dupuytren's compressor would in many, perhaps most, instances efficiently and more conveniently replace it. The use of the actual cautery is, we are sure, quite too much neglected by British surgeons. The extraordinary tendency of the cicatrix of a burn to contract is a familiar fact; whence we may appreciate the amount of contraction that may result from a *circular* cicatrix thus produced.

We always feel much hesitation in pronouncing any opinion respecting a point on which we have had no practical experience; but we must confess that we have detailed Dr. Dieffenbach's proceedings for the cure of a hernia concurrently with that of artificial anus, as a curiosity of operative surgery. When Dr. Dieffenbach published an account of his outrageous operation for stammering, by sub-mucous section of the base of the tongue, he said it was an operation not to be undertaken save by those endowed with the "operative temperament;" and we think the temperament in question should be possessed in very full measure, and, perhaps, coupled with the absence of some other qualities which we need not specify, to induce a surgeon to imitate the proceedings detailed at the commencement of our 35th page.

## Figures and Tables

**Fig. 1. Fig. 2. Fig. 3. f1:**